# D-dopachrome tautomerase contributes to lung epithelial repair via atypical chemokine receptor 3-dependent Akt signaling

**DOI:** 10.1016/j.ebiom.2021.103412

**Published:** 2021-06-04

**Authors:** Shanshan Song, Bin Liu, Habibie Habibie, Jelle van den Bor, Martine J. Smit, Reinoud Gosens, Xinhui Wu, Corry-Anke Brandsma, Robbert H. Cool, Hidde J. Haisma, Gerrit J. Poelarends, Barbro N. Melgert

**Affiliations:** a Groningen Research Institute of Pharmacy, Department of Molecular Pharmacology, University of Groningen, Antonius Deusinglaan 1, 9713 AV Groningen, the Netherlands; b Groningen Research Institute of Pharmacy, Department of Chemical and Pharmaceutical Biology, University of Groningen, Antonius Deusinglaan 1, 9713 AV Groningen, the Netherlands; c University Medical Center Groningen, Groningen Research Institute of Asthma and COPD, University of Groningen, Hanzeplein 1, 9713 GZ Groningen, the Netherlands; d Faculty of Pharmacy, Hasanuddin University, Makassar 90245, Indonesia; e Division of Medicinal Chemistry, Amsterdam Institute of Molecular and Life Sciences, Vrije Universiteit Amsterdam, De Boelelaan 1108, 1081 HZ Amsterdam, the Netherlands; f University Medical Center Groningen, Department of Pathology and Medical Biology, University of Groningen, Hanzeplein 1, 9713 GZ Groningen, the Netherlands

**Keywords:** MIF, emphysema, alveolar epithelial cells, type II cells, COPD, CXCR7

## Abstract

**Background:**

Emphysematous COPD is characterized by aberrant alveolar repair. Macrophage migration inhibitory factor (MIF) contributes to alveolar repair, but for its structural and functional homolog D-dopachrome tautomerase (DDT) this is unknown. MIF mediates its effects through CD74 and/or C-X-C chemokine receptors 2 (CXCR2), 4(CXCR4), and possibly 7 (ACKR3). DDT can also signal through CD74, but interactions with other receptors have not been described yet. We therefore aimed at investigating if and how DDT contributes to epithelial repair in COPD.

**Methods:**

We studied effects of recombinant DDT on cell proliferation and survival by clonogenic assay and annexin V-PI staining respectively. DDT-induced signaling was investigated by Western blot. Effects on epithelial growth and differentiation was studied using lung organoid cultures with primary murine or human epithelial cells and incubating with DDT or an ACKR3-blocking nanobody. DDT-ACKR3 interactions were identified by ELISA and co-immunoprecipitation.

**Findings:**

We found that DDT promoted proliferation of and prevented staurosporine-induced apoptosis in A549 lung epithelial cells. Importantly, DDT also stimulated growth of primary alveolar epithelial cells as DDT treatment resulted in significantly more and larger murine and human alveolar organoids compared to untreated controls. The anti-apoptotic effect of DDT and DDT-induced organoid growth were inhibited in the presence of an ACKR3-blocking nanobody. Furthermore, ELISA assay and co-immunoprecipitation suggested DDT complexes with ACKR3. DDT could activate the PI3K-Akt pathway and this activation was enhanced in ACKR3-overexpressing cells.

**Interpretation:**

In conclusion, DDT contributes to alveolar epithelial repair via ACKR3 and may thus augment lung epithelial repair in COPD.

**Research in Context**

**Evidence before this study**

COPD is associated with defective epithelial repair. MIF is a pleiotropic cytokine found to stimulate type II alveolar epithelial cell proliferation through one of its receptors CD74. Other known receptors for MIF are the C-X-C chemokine receptor 2, 4, and 7. DDT is a structural and functional homolog of MIF and DDT was also found to signal through CD74. Other receptors had not been identified yet and its role in lung tissue was unknown.

**Added value of this study**

Our study shows a new biological activity for DDT, i.e. stimulation of alveolar epithelial proliferation and prevention of apoptosis in these cells through ACKR3. Using murine and human lung organoids, we found that DDT treatment promoted alveolar organoid development and growth which could be inhibited by blocking ACKR3.

**Implications of all the available evidence**

Our study has uncovered that DDT contributes to proliferation of alveolar epithelial cells and alveolar growth via ACKR3. Importantly, this ability of DDT was retained in epithelial cells from patients with COPD providing a potential target for inducing alveolar repair in COPD.

Alt-text: Unlabelled box

## 1 Introduction

Chronic obstructive pulmonary disease (COPD) is one of leading causes of death worldwide and is characterized in many patients by emphysematous lung tissue destruction with impaired or insufficient alveolar epithelial repair [1]. Currently, there are no effective treatments able to reverse these structural defects apart from lung transplantation [2]. Notably, several novel pharmacological approaches to repair and regenerate lung epithelium have recently been suggested that may have the potential improve structural defects [3,4].

The human lung epithelium contains progenitor cells with repair and renewal capacities after injury. For example, basal cells in the distal airway and alveolar type II (ATII) cells in alveoli [5,6] have repair and renewal capacities [7]. ATII cells can self-renew and differentiate into alveolar type I (ATI) cells and are thought to be the key regenerative players in the lung [8]. Defective ATII regeneration can result in disturbed repair after damage and alveolar destruction which has a direct effect on gas exchange. Thus, pharmacologically targeting ATII to promote proliferation, differentiation and anti-apoptosis could be a potential therapy to restore/improve tissue repair in emphysematous lung tissue destruction in COPD. A candidate factor for this could be macrophage migration inhibitory factor (MIF), which has been shown to promote ATII proliferation through interactions with CD74 [9]. In addition, mice deficient for MIF were shown to spontaneously develop emphysema, emphasizing the importance of MIF for alveolar repair [10].

MIF is a pleiotropic immunomodulatory cytokine with many reported properties [11], [12], [13], [14]. CD74 is the main receptor of MIF and MIF-CD74 binding leads to activation of the ERK1/2 MAP kinase pathway [15] . MIF also shows chemokine-like activities via non-cognate interactions through binding to the C-X-C chemokine receptors - 2 (CXCR2) and 4 (CXCR4) [14]. In addition, MIF can bind to ACKR3 (in the past referred to as CXCR7) resulting in activation of the PI3K/Akt pathway, which enhances cell survival [16].

D-dopachrome tautomerase (DDT) is a structural and functional homolog of MIF and is also known as MIF2. It lacks the pseudo-(E)LR motif (Asp44-X-Arg11) and Arg-Leu-Arg (RLR) N-like-loop region, which allow MIF to bind to CXCR2 and CXCR4 [17], [18], [19]. Therefore, DDT has an attenuated ability to recruit neutrophils [20], but DDT seems to possess all other important properties and functions of MIF. For instance, DDT also binds to CD74 and stimulates the same ERK1/2 MAP kinase pathway [21]. Until now, CD74 is the sole known receptor for DDT and it is unknown whether DDT binds to ACKR3. In addition, the biological role of DDT in lung tissue has not yet been studied thus far.

Since the structure of DDT and its activities are highly similar to those of MIF, we hypothesized that DDT may play a hitherto unidentified role in lung epithelial repair. Therefore, we investigated the effects of DDT on epithelial repair in lung tissue and the involvement of ACKR3.

## 2 Materials and methods

### 2.1 Human lung tissue

Human lung tissue was used for isolation of epithelial cells for organoid cultures (n=10) and immunohistochemistry for DDT expression patterns (n=3). Histologically normal lung tissue was anonymously donated by individuals with COPD (n=10) or without COPD (n=3) undergoing surgery for lung cancer and not objecting to the use of their tissue. COPD patients included ex- and current- smoking individuals with GOLD stage I-IV disease (GOLD I=2, GOLD II=4, GOLD IV=4). Characteristics of patients can be found in Table 1. Subjects with other lung diseases such as asthma, cystic fibrosis, or interstitial lung diseases were excluded. The study protocol was consistent with the Research Code of the University Medical Center Groningen (UMCG) and Dutch national ethical and professional guidelines (www.federa.org). Sections of lung tissue of each patient were stained with a standard haematoxylin and eosin staining and checked for abnormalities by a lung pathologist.

**Table 1:** Patient characteristics (medians with range are presented).

	Control (n=3)	COPD (n=10)

Sex (male/female)	1/2	5/5
Age (years)	55 (53-77)	63 (56-71)
Smoking status	1 exsmoker/	1 nonsmoker/
	2 current smoker	6 exsmoker/
		2 current smoker/
		1 current/exsmoker
FEV_1_ (% predicted)	105 (105-112)	62 (15-107)
FVC (% predicted)	106 (103-117)	81 (39-129)
COPD GOLD stage	NA	2 GOLD stage I
		4 GOLD stage II
		4 GOLD stage IV

COPD = chronic obstructive pulmonary disease; FEV_1_ = Forced exhaled volume; FVC= forced vital capacity; NA= not applicable.

### 2.2 Mice

All animal experiments were performed after review by and approval from the Dutch National Animal Care and Use Committee according to strict governmental and international guidelines on animal experimentation (License number AVD10500202011285).

Female and male C57BL/6N mice were housed separately in the animal facility of the University Medical Center Groningen and provided with sterile rodent chow and water ad libitum. Experiments were performed with both female and male mice at 10-12 weeks of age.

### 2.3 Cloning and Purification of DDT protein

**Fig. 1:**
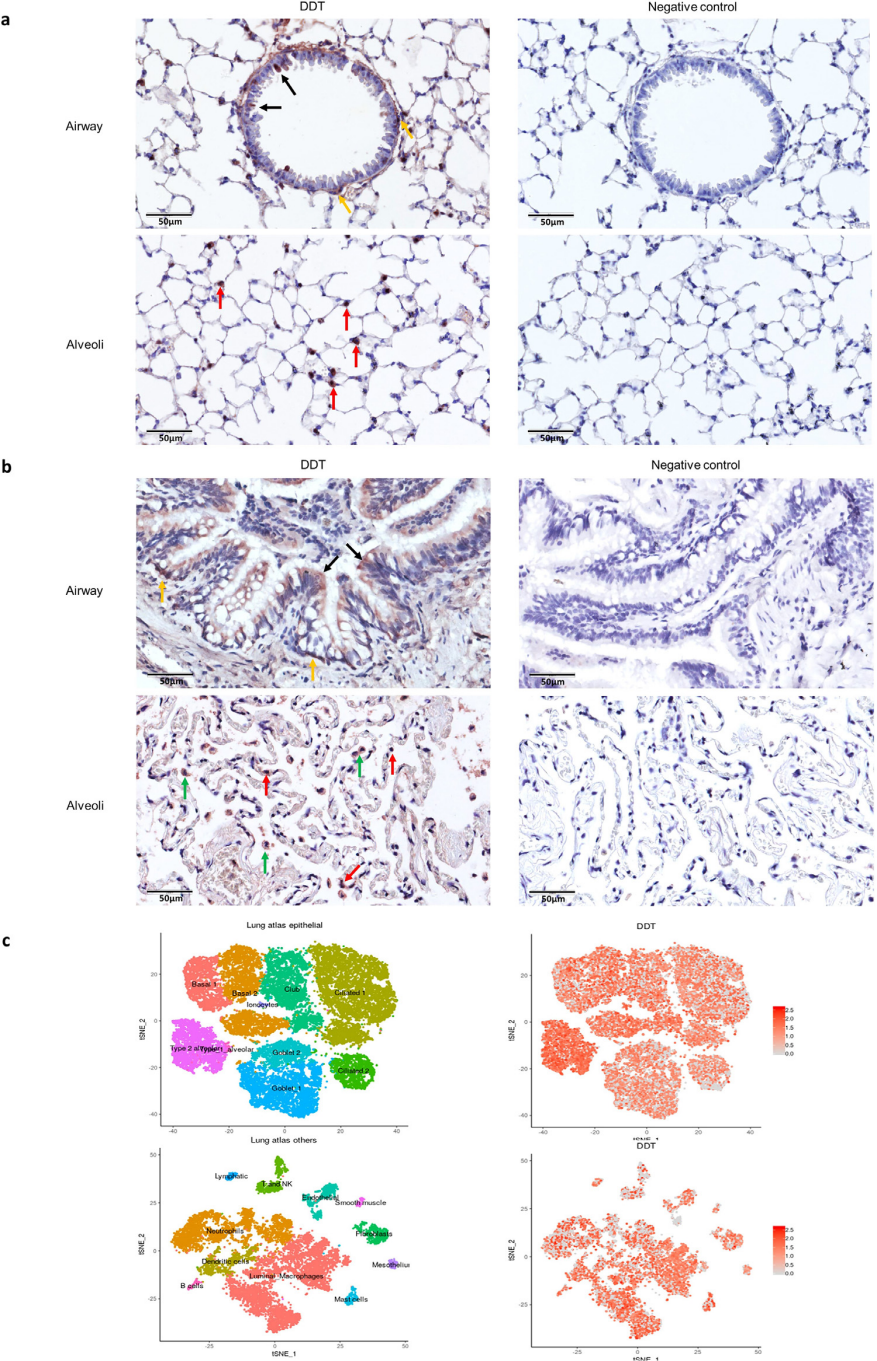
Immunohistochemical analysis of DDT expression in control lung tissue from a mouse and a patient with normal lung function. (a) Representative pictures of lung tissue from a healthy control mouse stained for DDT. Left panels: DDT expression was observed in ATII (red arrows), ciliated epithelial cells (black arrows) and basal epithelial cells (yellow arrow) as indicated by the arrows. Right panels: negative controls for airway and alveolar tissue. (b) Representative pictures of control lung tissue from a patient with normal lung function undergoing surgical resection for lung cancer. Left panels: DDT expression was observed in ATII (red arrows), macrophages (green arrows), ciliated cells (black arrows) and basal epithelial cells (yellow arrows) as indicated by the arrows. Right panels: negative controls for airway and alveolar tissue. (c) Left t-SNE shows the major epithelial, immune and mesenchymal clusters present in human lung tissue. Right t-SNE depicts DDT mRNA expression in different clusters in lung according to the Lung Cell Atlas. (For interpretation of the references to colour in this figure legend, the reader is referred to the web version of this article.)

Gene sequences of the human DDT gene and the murine DDT gene were adapted to bacterial expression and obtained from Invitrogen. After subcloning into a pET20b(+) expression vector, IPTG-induced expression was performed in *E.coli* strain BL21(DE3). Both human and murine DDT proteins were overproduced overnight at 20°C and harvested cells were resuspended in 20 mM diethanolamine, 2 mM dithiothreitol, 10% glycerol, pH 8•5; 3 mL/g of wet cells. Cells were disrupted using sonication and extracts were clarified by centrifugation for 60 min at 40,000xg and 4°C. The soluble fraction was purified using a Q sepharose column (GE Healthcare) with a gradient of NaCl. The fractions containing DDT were brought to 1•7 M ammonium sulfate and loaded on a phenyl sepharose column (GE Healthcare) and eluted with a gradient to 0 M ammonium sulfate in a 20 mM sodium phosphate buffer, pH 8•0. Finally, the proteins were purified by size exclusion chromatography on a Superdex75 column (GE Healthcare) in 20 mM sodium phosphate buffer, pH 8•0, with an elution volume characteristic for trimeric DDT. The collected protein was concentrated using a VivaSpin centrifugation column with a molecular weight cut off at 5000 D (Sartorius Stedim Biotech GmbH). Lipopolysaccharide (LPS) contamination was eliminated using a Pierce^TM^ high capacity LPS removal resin (ThermoFisher Scientific). LPS-free PBS (Millipore, Merck) was used for adjusting DDT concentration. Purified proteins were aliquoted, snap frozen in liquid nitrogen, and stored at -80°C. Protein concentrations were determined by Bradford assay using BSA as standard. Protein purity was verified by SDS-PAGE and the gels stained with Instant Blue (Expedeon) and silver staining (Supplemental data file 1 Fig. 1a). The protein's molecular size was confirmed by mass spectroscopy (Supplemental data file 1 Fig. 1b). Enzymatic activity of the protein was assessed by measuring the tautomerase activity using the substrate 4-hydrophenypyruvic acid (4HPP) and measuring a change in absorbance at 306 nm (Supplemental data file 1 Fig. 1c). Neither of the recombinant DDT proteins contained LPS contamination as they did not induce TNFα mRNA expression in RAW264.7 macrophages, whereas positive control LPS did (Supplemental data file 1 Fig. 1d).

### 2.4 Cell lines and culture conditions

A549 epithelial cells (RRID: CVCL_0023, ATCC CCL-185) were analyzed by the American Type Culture Collection using short tandem repeat analysis to validate their identity. A549 epithelial cells were cultured in DMEM (GibcoTM #31966-021), supplemented with 10% fetal bovine serum, 100 U/ml penicillin/streptomycin (GibcoTM#10378016) at 37°C with 5% CO_2_ in humidified air.

Murine lung fibroblasts (RRID: CVCL_0437, ATCC #CCL-206) were maintained in DMEM (Gibco, #3196-021) / Ham's F12 medium (Lonza, #BE12-615F) supplemented with 10% fetal bovine serum (FBS), penicillin/streptomycin (100 U/ml, Gibco #15140-122) and glutamine (1%, Life Technologies #35050–061). MRC5 human lung fibroblasts (ATCC #CCL-171) were maintained in Ham's F12 medium supplemented with 10% FBS, penicillin/streptomycin (100 U/ml) and glutamine (1%). Prior to organoid culture, CCL206 or MRC5 fibroblast growth was inactivated with mitomycin C (10 μg/ml, Sigma-Aldrich #M4287) for 2 h, followed by washing with PBS (GibcoTM #14190-094) three times and trypsinization.

Cells were tested monthly for mycoplasma contamination with the MycoSensor PCR Assay Kit (Agilent Technologies, #302108) and cells were only used when testing negative.

### 2.5 Development of a ACKR3-specifc nanobody (VUN702) via phage-display

Two llamas were immunized with ACKR3-encoding plasmid DNA as described previously [22]. Nanobody phage-display libraries were constructed according to the previously described method [23]. Briefly, selections for ACKR3-specific binders were performed using phage panning on ACKR3-expressing or empty (null) virus-like particles (Integral Molecular, Philadelphia, PA, USA) immobilized in MaxiSorp plates (Nunc, Roskilde, Denmark). Three consecutive rounds of selection were performed. The isolated ACKR3-binding nanobodies fused with His6 tags were purified onto Nickel Excel SepharoseTM column (GE Healthcare, Chicago, IL, USA) and eluted with a buffer containing 500 mM imidazole (Sigma-Aldrich, St. Louis, MO, USA). The elution buffer was exchanged for Dulbecco's phosphate buffered saline (DPBS; Thermo Fisher Scientific) by desalting using Amicon® Ultra-0.5 Centrifugal Filters with molecular weight cut-off of 3000 (Sigma-Aldrich).

### 2.6 Cell isolation and organoid culture

Murine primary lung epithelial cells were isolated and cultured as previously described with slight modifications [24,25]. Briefly, mice were sacrificed, cardiac perfusion was performed and lungs were injected with a dispase (BD Biosciences #354235) /agarose (Sigma-Aldrich #A9414) mixture. Subsequently, lung tissue without trachea was digested with a dispase/agarose mixture at room temperature for 45 min and was homogenized to a single cell suspension. Cells in suspension were negatively selected using a mix of mouse CD45-selecting (RRID:AB_2877061, Miltenyi #130–052-301) and mouse CD31-selecting (RRID:AB_2814657, Miltenyi #130–097-418) microbeads. Then CD45-CD31-negative cells were further positively selected with mouse Epcam-selecting microbeads (Miltenyi #130-105-958). EpCAM^+^ cells (10,000) and CCL206 fibroblasts (10,000) were resuspended in 100 μl DMED/F12 medium containing 10% FBS diluted 1:1 with growth-factor-reduced Matrigel (Corning #354230), and were seeded in a 24-well 0•4 μm Transwell insert (Falcon #353095).

Human primary lung epithelial cells were isolated from lung tissue from patients with or without COPD. Distal human lung tissue was dissociated and homogenized with a multi-tissue dissociation kit 1 (Miltenyi #130-110-201) using a GentleMACS Octo dissociator at 37°C (Miltenyi #130-096-427). The resulting suspension was negatively selected using a mix of human CD45-selecting (RRID:AB_2783001, Miltenyi #130–045-801) and human CD31-selecting (Miltenyi #130–091-935) microbeads. The CD45-CD31-negative cells were further positively selected with human EpCAM-selecting microbeads (RRID:AB_2832928, Miltenyi #130-061-101). EPCAM^+^ cells (5000) were seeded with MRC5 fibroblasts (5,000) in Matrigel.

After solidifying, organoid cultures were maintained in DMEM/F12 medium with 5% (v/v) FBS, 1% insulin-transferrin-selenium (Gibco #15290018), recombinant mouse EGF (0•025 µg/ml, Sigma, #SRP-3196), bovine pituitary extract (30 µg/ml, Sigma, #P-1476). To prevent dissociation-induced apoptosis, ROCK inhibitor (10µM, Y-27632, TOCRIS #1254) was added for the first 48 h. Organoid cultures from mouse lung tissue were treated with recombinant mouse DDT (rmDDT, 100 ng/ml). Organoids from human lung tissue were treated with recombinant human DDT (rhDDT, 100 ng/ml) or/and 1 μM of blocking nanobody against ACKR3 (VUN702). All treatments were freshly added to the cultures every two days. Organoid cultures were maintained at 37°C with 5% CO_2_ in humidified air. The number of organoids was manually counted and organoid diameter was measured at day 14 of the culture using an Olympus IX50 light microscope connected to Cell^A software (Olympus).

### 2.7 Colony formation assay

A549 epithelial cells were seeded at a density of 100 cells/well in six-well plates. On day 2, cells were treated with 50 ng/ml, 100 ng/ml or 200 ng/ml recombinant human DDT. After 11 days, colonies were fixed with 4% formaldehyde (Klinipath, #4078.9010) and stained with 0•25% (w/v) crystal violet for visualization. Colonies (>50 cells per colony) were counted. Each experiment was performed in triplicate and repeated eight times.

### 2.8 Apoptosis assay

A549 epithelial cells were seeded in 12-well tissue culture plates and were left untreated or pre-treated with rhDDT (100 ng/ml) or CXCL12 (100 ng/ml, PeproTech #300-28A) for 1 h. Afterwards cells were washed with PBS and were treated with 100 nM staurosporine (Sigma-Aldrich #S6942) for 24 h. In selected experiments, cells were pretreated with 1 μM of ACKR3-blocking nanobody VUN702 or a control nanobody VUN100 before adding DDT or CXCL12. After treatment, cells floating in the medium were collected and adherent cells were detached with 0•05% trypsin. Culture medium containing 10% fetal bovine serum was then added to inactivate trypsin and cells were centrifuged for 10 min at 300g. The supernatant was removed and cells were stained with Annexin V-APC and propidium iodide (PI, RRID: AB_2575165, eBioscience #88-8007) according to the instructions of the manufacturer. Unstained cells were used as a negative control. The cells were analyzed immediately after staining using a Cytoflex flow cytometer (Beckman Coulter, Woerden, the Netherlands). For each sample, 20,000 cells were counted and data were analyzed using FlowJo software (Tree start, Ashland, USA). Annexin V^+^PI^−^ cells were identified as early-stage apoptotic cells and double-positive cells as late-stage apoptotic cells.

### 2.9 Generation of EGFR knockout cell with CRISPR/Cas9 [**26**]

Two EGFR targeting CRISPR/Cas9 GFP knockout plasmids, each encoding the Cas9 nuclease and a 20-nucleotide guide RNA (gRNA) targeting exons 2 and 3 of the EGFR, were supplied as a combination pool (Santa Cruz Biotechnology, Dallas, TX, USA). A549 epithelial cells were transfected with 3 µg of CRISPR/Cas9 plasmids pool using Lipofectamine 3000 (ThermoFisher Scientific,#L3000008). After one day of transfection, cells were treated with 2 µg/ml of puromycin for three days. Single cell suspensions were seeded into 96-well plates for clonal expansion. Colonies were tested for EGFR knockout by Sanger sequencing, Western blot, and flow cytometry analysis.

### 2.10 Immunohistochemistry

**Fig. 2:**
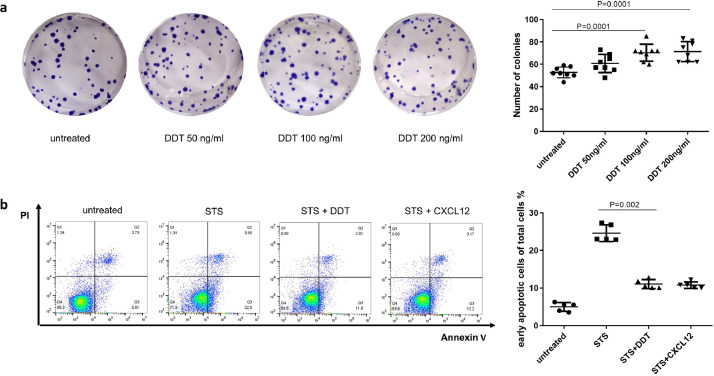
DDT promotes proliferation of A549 epithelial cells and prevents staurosporine-induced apoptosis. (a) A549 epithelial cells were incubated with 50 ng/ml, 100 ng/ml or 200 ng/ml of DDT for 11 d in a clonogenic assay. These increasing concentrations resulted in higher proliferation of A549 epithelial cells in a dose-dependent manner. Eight independent experiments were done. Groups were compared using a Kruskal–Wallis with Dunn's corrections test, p<0•05 was considered significant. (b) A549 epithelial cells were pre-treated with 100 ng/ml DDT or positive control CXCL12 (100 ng/ml) for 1 h prior to incubation with 100nM staurosporine (STS). Five independent experiments were done. Groups were compared using a Kruskal–Wallis with Dunn's correction for multiple testing, p<0•05 was considered significant.

Immunohistochemical analysis of DDT expression was performed on 3 µm-sections of paraffin embedded mouse lung tissue and human lung tissue. Sections were deparaffinized with xylene and rehydrated with PBS. Antigen-retrieval was performed by incubating sections from mouse lung tissue in 10 mM citric acid buffer (pH=6) or sections from human lung tissue in 0•1 M Tris-HCl buffer (pH=9) at 80 °C for 2 h. Thereafter, an antibody against human/murine DDT (RRID:AB_2614523, Rockland, #600-401-R05) was applied in a dilution of 1:50 for 1 h at room temperature. Afterwards, the sections were washed with PBS three times and further incubated with a goat anti-rabbit IgG(H+L) HRP-conjugated secondary antibody (RRID:AB_2795950, Southern Biotech, #4049-05, 1:200) for 30 min at room temperature. DDT staining was visualized by ImmPACTRNovaREDTM (RRID:AB_2336522, Vector, #SK-4805). Subsequently, the tissue was counterstained with haematoxylin (Klinipath BV, #4085.9002). After dehydration, sections were covered with a coverslip using Depex (SERVA, #18243). The specificity of the anti-DDT antibody for DDT was confirmed with human and murine DDT and MIF by western blot (Supplemental data file 1 Fig. 2).

### 2.11 ELISA

We examined the binding between ACKR3 and DDT by ELISA assay, coating plates with either rhDDT or rhMIF (positive control) and then incubation with HeLa cell lysate, which highly expresses ACKR3, followed by an antibody against ACKR3. Coating plates with HeLa cell lysate was included as another positive control. In short, high-binding 96-well plates (Greiner, #M4561) were coated with rhDDT (100 μL, 1 µM, 100 nM, 10 nM), rhMIF (1 uM), or Hela cell lysate (RRID:CVCL_0030, ATCC CCL-2) in 0•1M NaHCO_3_ (Merck, #S8875, pH 8•6) buffer overnight at 4 °C. This was followed by washing with TBST (0•05% v/v Tween 20 in PBS) and blocking of aspecific binding with 2% w/v bovine serum albumin (BSA, Sigma, #A2153) in PBS for 2 h. After washing three times with TBST, Hela cell lysate (ATCC CCL-2) was added and incubated at 4°C for 20 min. Plates were then washed with TBST and incubated with a primary antibody against ACKR3 (1:1000, RRID:AB_1240639**,** GeneTex #GTX100027). After washing with TBST, a goat anti-rabbit HRP-conjugated secondary antibody (1:1000, RRID:AB_2617138, DAKO, p0448) was added and incubated for 1 h. Binding was visualized using a One‐step Turbo TMB substrate solution (ThermoFisher Scientific, #34022) and stopped by adding 100 μL of 1 M sulfuric acid solution. The absorbance was measured at 450 nm. To assess background binding, we also included four negative controls: 1. incubation without HeLa lysate to show a specific binding between rhDDT and the primary antibody; 2. incubation without the primary antibody against ACKR3 to show aspecific binding between rhDDT and the secondary antibody; 3. coating of the well with BSA to show background given by BSA; 4. coating of the wells with lysis buffer to show background given by lysis buffer.

### 2.12 Immunoprecipitation

5 µg anti‐DDT antibody (Rockland, #600-401-R05), anti-CXCR7 antibody (GeneTex, #GTX100027), or IgG isotype control antibody (Invitrogen, #10500C) were covalently coupled to M‐270 Epoxy beads (Dynabeads Co-Immunoprecipitation kit, Thermofisher Scientific, #14321D) at 37°C for 20 h. The next day, 10^7^ A549 epithelial cells were incubated with 1 μg/mL rhDDT in complete medium at 4°C to prevent ligand receptor complex internalization. After washing with PBS, cell lysates were prepared and centrifuged to obtain nuclei-free lysates and these were then co‐immunoprecipitated for 30 min with the pre-coated beads at 4°C. Beads were washed three times with extraction buffer A and one time with 1xLWB supplied with the kit. The bead-bound proteins were then eluted using SDS loading buffer (50 mm Tris‐HCl, pH 6•8, 2% SDS, 6% glycerol) and visualized by western blot.

### 2.13 Western Blot

A549 epithelial cells were treated with 100 ng/ml rhDDT for 15 mins, 30 mins or 2 h. Cell lysates were made as previously described [26] and loaded onto a 4-15% Bis-Tris gel (Bio-Rad Laboratories, #4561084). Proteins were separated at 100V and subsequently transferred to polyvinylidene fluoride (PVDF) membranes (Roche, #03010040001). The membranes were blocked with 5% nonfat dry milk (BIO-RAD, #1706404) and incubated overnight at 4°C with one of the following primary antibodies: phospho-Akt (1:1000, RRID:AB_ 329828, Cell Signalling Technology, #9275S), Akt (1:1000 Cell Signalling Technology #4685S), phospho-ERK1/2 (1:1000, RRID:AB_331772, Cell Signalling Technology #4376S), ERK1/2 (1:1000, RRID:AB_330744, Cell Signalling Technology #9102S), phospho-BAD (1:500, RRID:AB_10547878 Cell Signalling Technology #4366S) or β-actin (1:10000, RRID:AB_10950489, Cell Signalling Technology, #8457). The membranes were further incubated with a goat anti-rabbit HRP-conjugated secondary antibody (1:2000, RRID:AB_2617138, DAKO, p0448) for 1 h at room temperature. For detection, blotted proteins were visualized with an ECL^TM^ Prime Western Blotting System (GE Healthcare #RPN2232). All expression levels were normalized to β-actin expression.

### 2.14 Immunofluorescence analysis

Lung organoids were stained for acetylated α-tubulin and prosurfactant protein C to identify ciliated airway epithelial cells and ATII cells respectively, as described previously by Ng-Blichfeldt et al. [24,25]. In short, organoids were fixed with ice-cold acetone/methanol (1:1 v/v) and unspecific staining was blocked with 5% BSA (w/v, Sigma #A2153). Organoids were then incubated with primary antibodies against acetylated α-tubulin (Santa Cruz Biotechnology #sc-23950) and prosurfactant protein C (MERCK #3194602) in a 1:200 dilution in PBS with 0•1% (w/v) BSA and 0•1% (v/v) Triton-X100 (Thermo Fisher #85111) at 4 °C overnight. Organoids were washed three times with PBS and incubation with the secondary antibodies (1:200, Donkey anti-Rabbit AlexaFluor 488, RRID:AB_2535792, Invitrogen #A21206; 1:200, Donkey anti-Mouse AlexaFluor 568, Invitrogen #A10037) was performed for 2 h at room temperature. Organoids were washed with PBS three times again and then cut from inserts and transferred onto glass slides with mounting medium containing DAPI (Abcam #104139) and a coverslip. Images were obtained by using a Leica DM4000b fluorescence microscope connected to Leica Application Suite software.

### 2.15 Statistics

All statistics were performed with GraphPad Prism 8•0. Mice used for isolation of lung cells were randomly selected from a population. Sample size for analyses was at least 5 and more if feasible, except for Western blot analyses which were done in triplicate when feasible. For datasets n<7 nonparametric testing was used. For datasets n≥7, a Shapiro-Wilk test was used to determine the normality of the data. For normally distributed data, parametric testing was used and otherwise data were log-transformed or nonparametric testing was used. For nonparametric testing between two groups a Mann Whitney U test was used for unpaired or a Wilcoxon test for paired data. For parametric testing between two groups a paired or unpaired Student t-test was used for paired or unpaired data respectively. For comparison of multiple-groups, a Kruskall wallis or Friedman test was used for nonpaired or paired nonparametric data respectively with Dunn's correction for multiple testing. For parametric data paired or nonpaired one-way ANOVA was used with Holm-Sidak's correction for multiple testing. Western blot data investigating different time points were compared using a repeated measure ANOVA and data investigating addition of DDT, the ACKR3-blocking nanobody, or both were compared using 2-way ANOVA. Data are presented as median ± range and p-values <0•05 were considered significant.

### 2.16 Role of funding source

The funder has had no role in study design, data collection, data analyses, interpretation of data, or writing of the report.

## 3 Results

### 3.1 DDT is expressed by ATII cells in lung tissue

To identify which cells in lung tissue express DDT, we did immunohistochemical analysis for DDT in lung tissue from healthy mice and humans with normal lung function undergoing surgical resection for lung cancer. We found DDT mainly expressed by epithelial cells, in particular by ATII cells in both mice (Fig. 1**a**) and human (Fig. 1**b**) lung tissue. In human control lung tissue, macrophages were also a major source of DDT (Fig. 1**b**). Furthermore, the newly generated Lung Cell Atlas (https://asthma.cellgeni.sanger.ac.uk/) showed DDT mRNA was expressed by many cells in human lung tissue and most abundantly in ATII cells and macrophages (Fig. 1**c**, picture generated from the atlas), which was consistent with our protein staining data.

### 3.2 DDT promotes A549 epithelial cell proliferation and protects them from apoptosis

Since ATII cells are progenitor cells that can repair damaged alveoli, we investigated the proliferative potential of DDT on ATII cells. Using a clonogenic assay with the ATII cell line A549, we found that increasing concentrations of rhDDT induced more proliferation in a dose-dependent manner as compared to untreated controls (Fig. 2**a**).

This effect on proliferation may be explained (in part) by decreased apoptosis as DDT's structural and functional homolog MIF was shown to rescue cells from apoptosis through binding to ACKR3 [16]. Therefore, we investigated whether DDT had similar effects on cell survival. A549 epithelial cells were pretreated with rhDDT or CXCL12 as a positive control for ACKR3 stimulation for 1 h before staurosporine was added to induce apoptosis. Upon pre-incubation with rhDDT or CXCL12, staurosporine-induced early apoptosis (Annexin V+/PI-) was significantly attenuated (Fig. 2**b**).

### 3.3 DDT activates ERK MAP kinase and the PI3K/Akt pathway

**Fig. 3:**
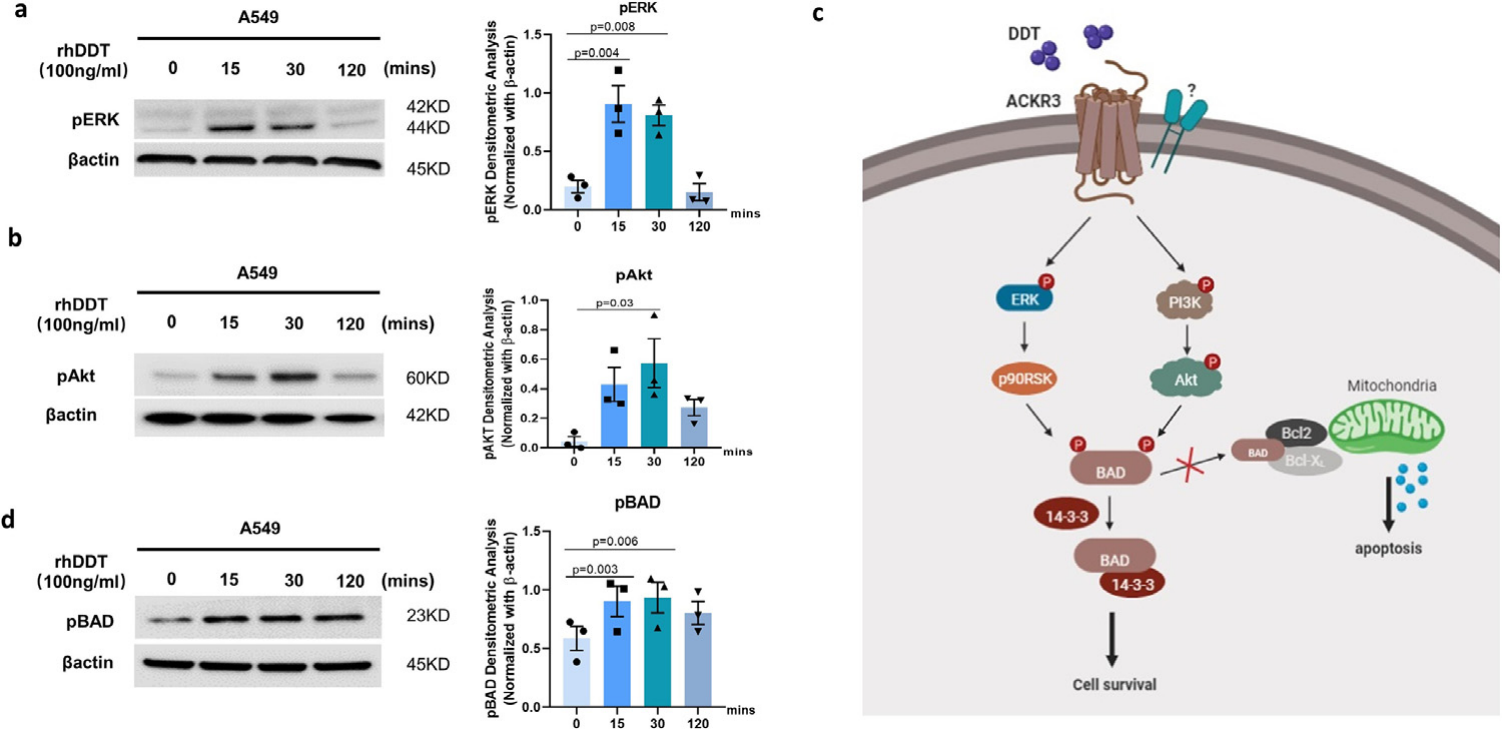
DDT activates the ERK-MAP kinase and PI3K-Akt pathways. A549 epithelial cells were treated with 100 ng/ml rhDDT for different time periods. (a, b, d) Cell lysates were analyzed for phosphorylation of ERK (p-ERK), Akt (p-Akt), and downstream BAD (p-BAD) by Western blot (n=3, original images can be found in supplemental data file 2). Beta-actin was used as a loading control. Groups were compared using repeated measures ANOVA, p<0•05 was considered significant. (c) Proposed model of DDT/ACKR3 induced signaling pathway. Created with BioRender.com

**Fig. 4:**
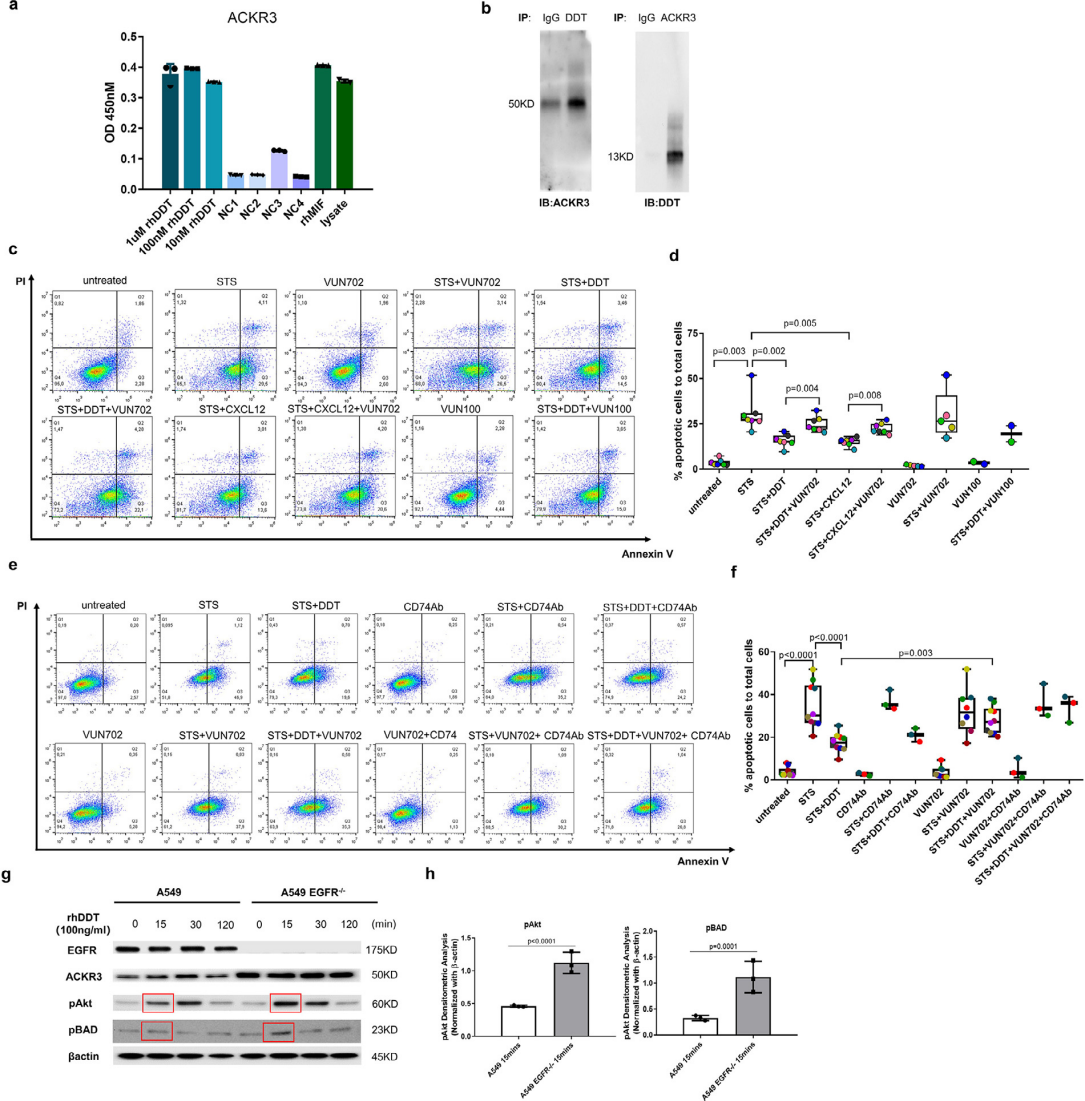
DDT mediates anti-apoptotic effects in A549 cells through ACKR3. (a) Quantification of binding of DDT to ACKR3-expressing cells as measured by ELISA. Wells were coated with DDT and binding of ACKR3-containing HeLa cell lysate was similar to wells coated with MIF or with ACKR3-containing HeLa cell lysate directly. Negative controls (NC) assessed unspecific binding between rhDDT and the primary antibody (no ACKR3-containing HeLa cell lysate added, NC1), unspecific binding between ACKR3 and the secondary antibody (no primary antibody added, NC2), BSA coating added instead of rhDDT (NC3), and lysis buffer added instead of HeLa cell lysate (NC4). Each experiment was performed in triplicate and three independent experiments were done. (b) Immunoprecipitation followed by western blot showed co-immunoprecipitation of DDT with ACKR3 (50KD) from A549 epithelial cell lysates (original images can be found in supplemental data file 2). IB: immunoblotting done with an antibody against either ACKR3 or DDT. IP: immunoprecipitation done with an antibody against either ACKR3 or DDT. IgG nonspecific antibody control. (c,d) A549 epithelial cells were pretreated with 1 μM of a ACKR3-blocking nanobody VUN702 or control nanobody VUN100 prior to treatment with 100 ng/ml DDT or 100 ng/ml CXCL12. Then cells were treated with staurosporine for 24 h. Seven independent experiments were done for experiments with VUN702. Two independent experiments were done for experiments with VUN100. Each color designates one independent experiment. Groups were compared using one-way ANOVA with Holm–Sidak's correction for multiple testing, p<0•05 was considered significant. (e,f) A549 epithelial cells were pretreated with 20 µg/ml of a neutralizing antibody against CD74 or/and 1 μM of ACKR3-blocking nanobody VUN702 prior to treatment with 100 ng/ml DDT. Then cells were treated with staurosporine for 24 h. Three independent experiments were done. Each colour designates one independent experiment. (g,h) Wild type and EGFR^-^^/-^ A549 epithelial cells were treated with 100 ng/ml DDT for different time periods. Cell lysates were analyzed for phosphorylation of Akt and BAD by Western blot (n=3, original images can be found in supplemental data file 2)). Beta-actin was used as a loading control. Groups were compared using a repeated measures ANOVA and graphs in f. only show quantification of the 15 mins time point. p<0•05 was considered significant.

To elucidate which signaling pathways are involved in DDT-induced proliferation and survival, we stimulated A549 epithelial cells with rhDDT for different time periods and analyzed phosphorylation of ERK1/2 and Akt. Interestingly, treatment with DDT was found to result in time-dependent phosphorylation of ERK1/2 (Fig. 3**a**) and Akt (Fig. 3**b**), whereas treatment with the vehicle alone did not activate ERK1/2 and Akt (Supplemental data file 1 Fig. 3). Akt phosphorylation can lead to phosphorylation-mediated inactivation of BAD which will promote cell survival (Fig. 3**c)**. When BAD is phosphorylated, it forms a heterodimer with the 14-3-3 protein and this prevents BAD from forming a heterodimer with B-cell lymphoma 2 (Bcl-2) or B-cell lymphoma-extra large (Bcl-xL). Association of BAD with Bcl-2 or Bcl-xL triggers cytochrome C release by mitochondria and subsequently induces apoptosis and BAD phosphorylation inhibits this [27,28]. Therefore, we also investigated whether DDT treatment leads to phosphorylation of BAD as an explanation for the anti-apoptotic effects observed. Our results clearly show that DDT treatment resulted in phosphorylation of BAD in a time-dependent manner (Fig. 3**d**). It is unlikely these results can be explained by an interaction of DDT with CD74 as expression of CD74 in A549 epithelial cells was extremely low (Supplemental data file 1 Fig. 4). Since the anti-apoptotic effects were comparable to those of CXCL12, which is the cognate ligand for ACKR3 [29,30], we subsequently investigated whether DDT could mediate its effect via ACKR3 as well.

### 3.4 DDT mediates its anti-apoptotic effects through ACKR3 in A549 epithelial cells

**Fig. 5:**
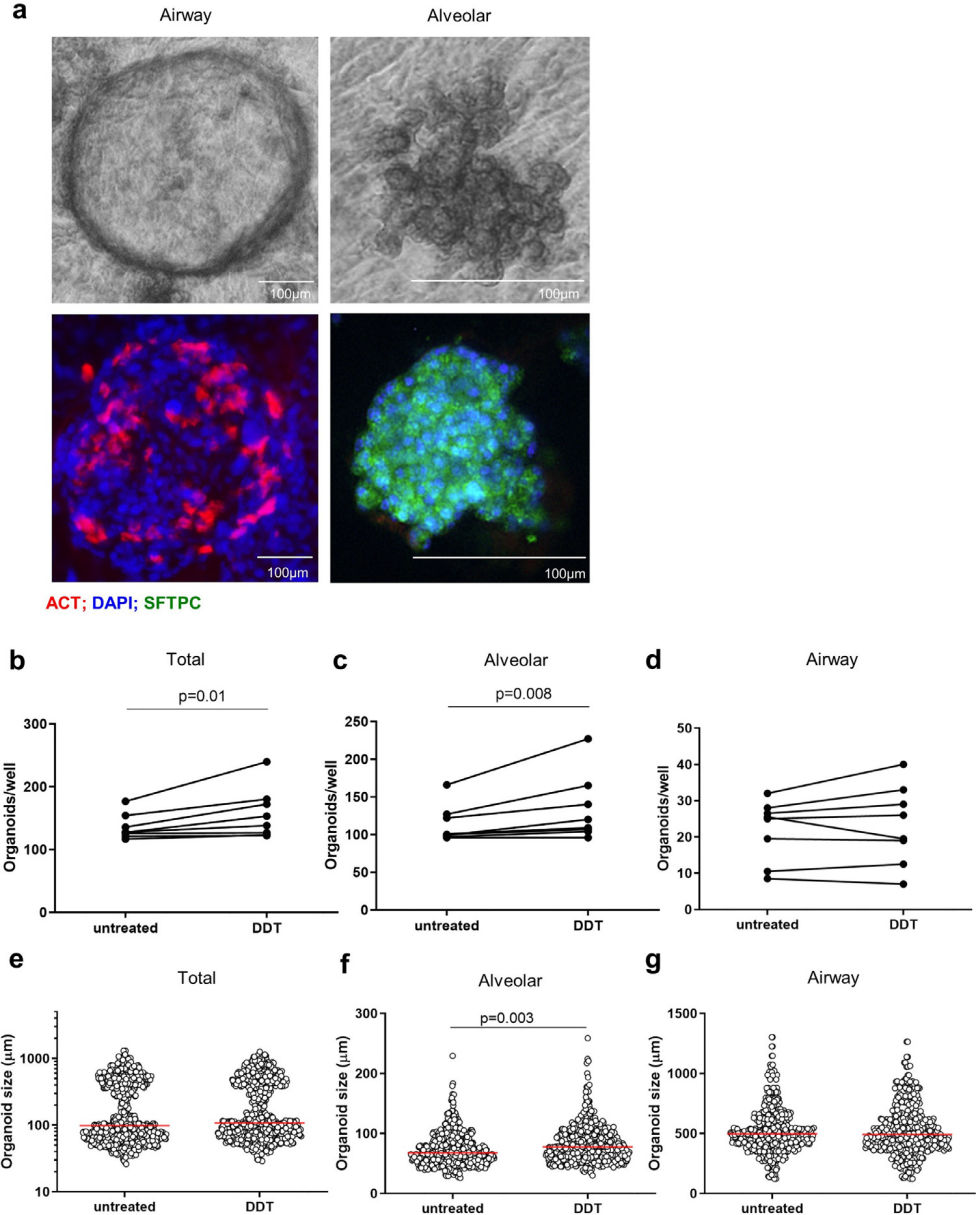
DDT promotes growth of murine alveolar organoids. (a) Light microscopy images of mouse airway and alveolar organoids morphologies and immunofluorescence images of mouse airway and alveolar organoids stained for acetylated tubulin (ACT, red), pro-surfactant protein C (SFTPC, green), and DAPI (blue). (b-d) Quantification of total, alveolar, and airway organoid numbers and (e-g) sizes at day 14 following treatment with or without DDT (100 ng/ml). Lung tissue was obtained from eight healthy mice. Groups were compared using a paired t test, p<0•05 was considered significant. (For interpretation of the references to colour in this figure legend, the reader is referred to the web version of this article.)

We examined the binding between ACKR3 and DDT by ELISA assay. We used rhMIF and HeLa lysate as positive controls as rhMIF has been shown before to bind to ACKR3 [16,31] and HeLa cells highly express ACKR3 [32]. We found DDT to have similar binding to ACKR3 as MIF while the negative controls showed low signal. The positive control of HeLa cell lysate had a high signal showing the assay worked (Fig. 4**a**). Although DDT lacks the pseudo(E)LR (Arg11,Asp44) motif and Arg-Leu-Arg (RLR) N-like-loop region that mediate binding between MIF and CXCR2 or CXCR4 respectively [14,19], we did check whether DDT could bind to CXCR4 using a similar ELISA set up. As expected, we did not find this to be the case (Supplemental data file 1 Fig. 5). Our ELISA data indicated that DDT can interact with ACKR3, which was also confirmed by co-immunoprecipitation. Co-immunoprecipitation of DDT and ACKR3 in A549 cell lysates further confirmed that DDT can form a complex with ACKR3 (Fig. 4**b**).

**Fig. 6:**
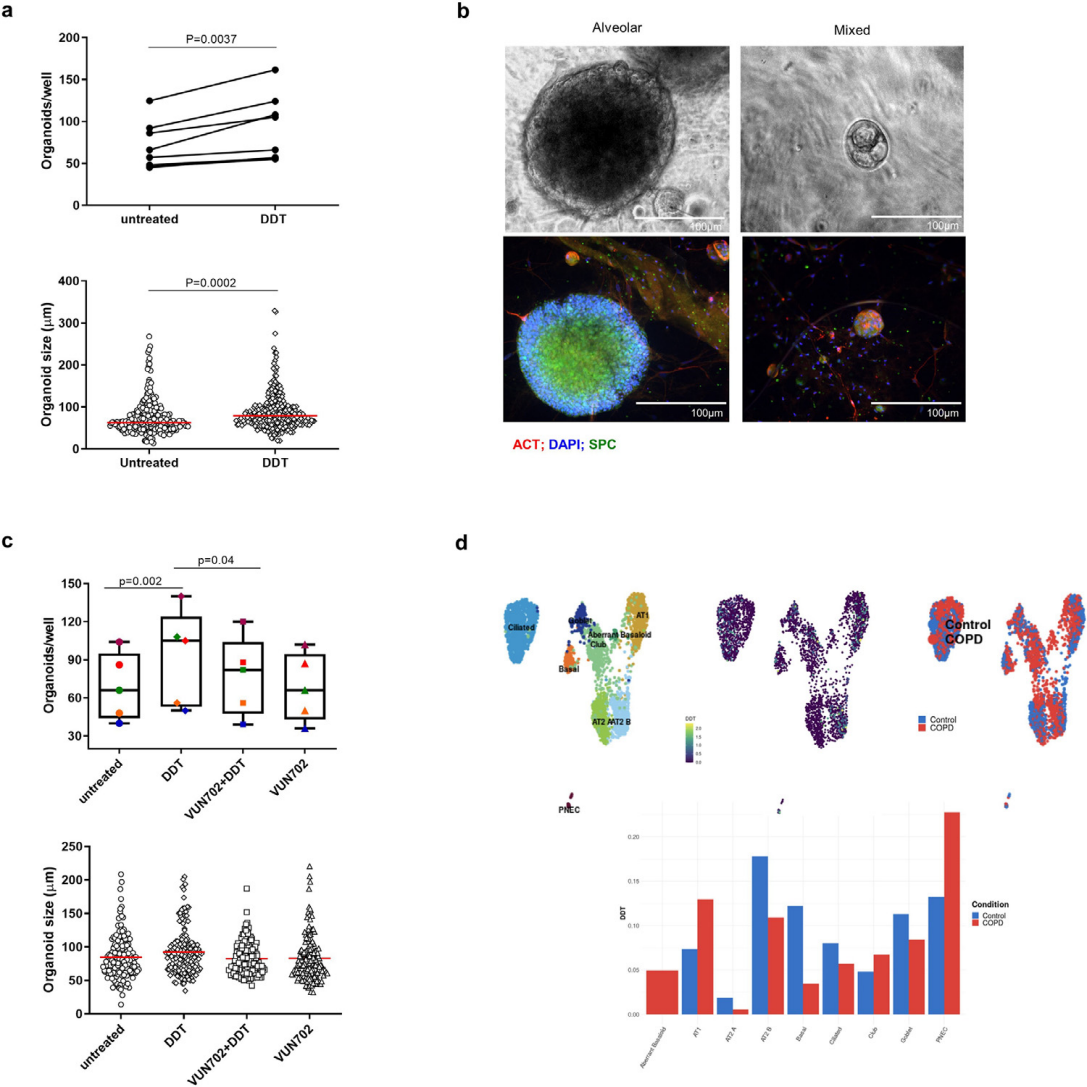
DDT promotes growth of alveolar organoids from lung tissue of COPD patients. (a) Quantification of the total number of human organoids (alveolar and mixed) and their size on day 14 following treatment with and without rhDDT (100 ng/ml). Lung tissue was obtained from eight patients with COPD GOLD stage I-IV. Groups in panel a were compared using a paired t test, p<0.05 was considered significant. (b) Upper panels: light microscopy images of human alveolar and mixed organoid morphologies; lower panels: immunofluorescence images of human alveolar and mixed organoids stained for acetylated tubulin (ACT, red), pro-surfactant protein C (SFTPC, green), and DAPI (blue). (c) Quantification of the total number of organoids (alveolar and mixed) on day 14 following treatment with and without rhDDT (100 ng/ml) or/and the ACKR3-blocking nanobody VUN702 (1 μM). Lung tissue was obtained from five patients with COPD GOLD stage I-IV. Each color designates one patient. Groups in panel a were compared using a Friedman test with Dunn's correction for multiple testing, p<0•05 was considered significant. Groups in panel c were compared using a two-way ANOVA, p<0•05 was considered significant. (d) Upper panel: UMAPs of DDT gene expression in different types of epithelial cells from lung tissue of patients with COPD or control donors. UMAPs are color labelled by cell type (*upper left*), DDT expression (*upper middle*), and disease status (*upper right*). Lower panel: Comparison of DDT gene expression in different types of lung epithelial cells from lung tissue of patients with COPD or control donors. Data taken from the COPDcellatlas.com. ATI= alveolar type I cells; ATII= alveolar type II cells; PNEC= pulmonary neuroendocrine cells. (For interpretation of the references to colour in this figure legend, the reader is referred to the web version of this article.)

It has previously been reported that MIF-dependent internalization of ACKR3 promotes survival via PI3K/Akt activation [16,33]. Therefore, we investigated whether ACKR3 was mediating the anti-apoptotic effects of DDT. CXCL12, the ligand for ACKR3, was used again as a positive control. Staurosporine was used to induce apoptosis and both DDT and CXCL12 could prevent A549 epithelial cells from apoptosis (Fig. 4**c** and 4**d**). However, in the presence of a blocking nanobody against ACKR3 (VUN702), DDT failed to prevent apoptosis in A549 epithelial cells. Similar effects were found for the positive control CXCL12 in combination with the ACKR3-blocking nanobody. VUN702 alone had no effects on staurosporine-induced-cell apoptosis. In addition, as a negative control we also included a nanobody (VUN100) targeting the viral chemokine receptor US28, which is not expressed in A549 epithelial cells and does not bind ACKR3. We found that VUN100 did not block the ability of DDT to prevent apoptosis. Furthermore, we also investigated whether CD74 was involved in DDT-mediated rescue from apoptosis using a neutralizing antibody against CD74. Treatment with this CD74 antibody did not inhibit DDT-mediated rescue from apoptosis and did not enhance the effects of the ACKR3 nanobody VUN702 (Fig. 4**e** and 4**f**). The isotype control (IgG_1Ƙ_) and nanobody control had no effects on apoptosis (Supplemental data file 1 Fig. 6a). Treatment with the CD74 antibody also did not inhibit DDT-induced Akt phosphorylation (Supplemental data file 1 Fig. 6b). To further confirm that DDT can activate PI3K/Akt signaling via ACKR3, we used an A549 EGFR knockout (EGFR^−/−^) cell line which we previously showed to have higher ACKR3 expression compared to A549 wild type epithelial cells [26]. DDT treatment of EGFR^−/−^ epithelial cells resulted in enhanced phosphorylation of Akt and BAD (Fig. 4**g** and 4**h**) compared to DDT treatment of A549 wild type epithelial cells. In addition, pretreatment of A549 wild type cells with VUN702 dose-dependently prevented the DDT-induced phosphorylation of Akt (Supplemental data file 1 Fig. 7a), which control nanobody VUN100 did not do (Supplemental data file 1 Fig. 7b).

We also investigated whether DDT binds directly to ACKR3 similar to CXCL12, using the displacement of CXCL12-AF647 from Nluc-tagged ACKR3 in A549 cells. Interestingly, preliminary data show both DDT and MIF were not able to displace CXCL12 from ACKR3, which suggests that DDT and MIF do not have the same binding site as CXCL12 (Supplemental data file 1 Fig. 8).

### 3.5 DDT promotes growth of mouse alveolar organoids

Our *in vitro* data indicated that DDT is involved in A549 epithelial cell proliferation and survival. Since this cell line may be different from primary lung epithelial cells, we investigated whether DDT has similar effects on primary epithelial cells using a model of lung organoids, which recapitulates critical features of lung epithelium [34], [35], [36]. Murine organoids were treated with rmDDT from the start of the culture until 14 days of culture. We counted the number of organoids and measured their diameter at day 14. Airway and alveolar organoids exhibited distinct morphologies at day 14 as shown in Fig. 5**a**. To confirm these different types of organoids, we performed immunofluorescence staining for acetylated alpha-tubulin to identify ciliated airway epithelium and for prosurfactant protein C to identify ATII alveolar epithelium (Fig. 5**a**).

Treatment with rmDDT resulted in significantly more organoids in total (Fig. 5**b**) and more alveolar-type organoids (Fig. 5**c**) than untreated controls and had no effect on airway-type organoids (Fig. 5**d**). In addition, DDT treatment also resulted in significantly bigger alveolar-type organoids while it had no effect on the size of total or airway-type organoids (Fig. 5**e-g**). Taken together, these results indicate that DDT promotes proliferation and differentiation of primary murine ATII cells.

### 3.6 DDT promotes growth of alveolar organoids from patients with COPD

It is currently unclear if loss of alveolar tissue in emphysema is due to innate defects in epithelial repair or not. To investigate if DDT can still induce epithelial growth in cells from lung tissue of COPD patients, we cultured organoids from cells isolated from lung tissue obtained from eight patients with GOLD Stage I-IV COPD. Similar to our findings in mice, we found more and larger organoids with DDT treatment compared to untreated controls when we quantified total organoids (Fig. 6**a**). In contrast to the mouse organoids, it was less clear by eye what type of structure they developed into, i.e. airway or alveolar. Immunofluorescent staining for acetylated alpha-tubulin and prosurfactant protein C indicated these organoids were either alveolar or a mixture of airway and alveolar types (Fig. 6**b**). No dedicated airway organoids were observed. Therefore, no further subdivision was made. These results indicate that DDT can promote growth of alveolar epithelial cells from lung tissue of COPD patients. In addition, adding the ACKR3-blocking nanobody showed that the effect of DDT on organoid growth could be inhibited through ACKR3 (Fig. 6**c**). The ACKR3-blocking nanobody alone did not affect organoid growth. These results again indicate that ACKR3 is involved in DDT-mediated epithelial cell survival/proliferation.

To investigate how DDT expression changes in COPD, we used the newly published COPDcellatlas.com to study DDT gene expression patterns in healthy and COPD-affected lung tissue (MedRxiv 2020:2020.09.13.20193417.). Similar to the results for the Lung Cell Atlas in figure 1, this database also shows that DDT is expressed in many epithelial cells and has the highest expression in ATII cells (Fig. 6**d**). Interestingly, DDT is especially expressed in ATIIB cells which resemble mature ATII cells with lower expression of progenitor genes and higher expression of canonical ATII cell markers such as surfactant protein C and A. DDT is also expressed in ATIIA cells that are WNT-responsive ATII epithelial progenitor cells, but to a lower extent. In both types of ATII cells, however, DDT is expressed at lower levels in patients with COPD compared to control.

## 4 Discussion

In this study we investigated whether DDT plays a hitherto unidentified role in lung tissue. Our findings demonstrate that DDT is expressed in lung tissue, contributes to alveolar growth via ACKR3 to promote proliferation of ATII cells and/or protects them from apoptosis. Importantly, DDT was also able to do this in epithelial cells from patients with COPD. This exciting novel function of DDT may therefore be further exploited to investigate therapeutic strategies aiming at stimulating lung tissue repair in diseases like COPD.

We have shown by immunohistochemistry and data from the human lung cell atlas that ATII cells are a predominant source of DDT in control mouse and human lung tissue. A recent study from our group has shown that DDT mRNA expression is higher in lung tissue of patients with COPD than in lung tissue of control patients [37]. These findings suggest that DDT is activated as a repair response to damage in lung tissue. This notion was confirmed by our findings that DDT promotes cell proliferation and/or survival through ERK1/2, Akt and BAD phosphorylation in A549 cells. ATII cells are progenitor cells that can self-renew and differentiate to ATI cells in damaged tissue and thereby maintain and repair alveoli after the injury. However, during lung injury, ATIIs may become apoptotic and will then be phagocytosed by macrophages [38]. As a consequence they lose the ability to renew and differentiate into ATI cells, leading to defective repair of lung tissue [39,40]. Therefore, DDT-mediated rescue from apoptosis could help in tissue repair in COPD. However, using the newly published COPDcellatlas.com we found lower DDT mRNA expression in ATII cells from patients with COPD compared to control. The higher expression of DDT in COPD lung tissue we recently reported may be caused by a compensatory increased expression in other, abundantly present, cell types such as ATI and club cells from the epithelial compartment and B cells and monocytes from the immune compartment (as can be seen on COPDcellatlas.com). How this variation in cell-specific DDT expression affects ATII proliferation and differentiation to ATI cells and alveolar repair in general needs to be elucidated further.

To the best of our knowledge, the only reported receptor for DDT was CD74. We have now shown that DDT can interact with ACKR3 and can also exert clear effects on cell signaling and cell behavior through ACKR3. DDT significantly prevented apoptosis to a similar degree as CXCL12, a known ligand of ACKR3 [41,42], and in both cases their effects were inhibited by a blocking nanobody against ACKR3. Previous studies have shown that CXCL12 binding to ACKR3 can induce cell proliferation and survival through PI3K/Akt pathway [31,43], as we have now shown for DDT. Using organoids from tissue of COPD patients we also found that DDT-induced growth of organoids was blocked by the ACKR3-blocking nanobody. Taken together, this strongly suggests that DDT exerts anti-apoptotic effects and promotes organoid growth through ACKR3.

Some recent reports have shown that ACKR3 is highly expressed in lung tissue and that the ACKR3 agonist TC14012 can decrease collagen I deposition and can protect the alveolar epithelial structure and function in a mouse model of bleomycin-induced lung fibrosis [44,45]. These data, together with ours, make a strong case for DDT-ACKR3-mediated lung epithelial repair. However, our preliminary data showed that both DDT and MIF were not able to displace CXCL12 from ACKR3, which suggests that DDT and MIF do not have the same binding site as CXCL12. Two hypotheses for this finding are worth further exploring. Firstly, DDT may bind to ACKR3 directly but the binding site is different from CXCL12. Secondly, ACKR3 may form heterodimers with CD74 or another unknown receptor of DDT that influence downstream signaling of ACKR3. Chemokine receptors are well known to form heterodimeric complexes. For example, MIF was found to engage ACKR3/CD74 and ACKR3/CXCR4 to activate MAPK ERK signaling [31]. However, interactions with CD74 are unlikely as we did not find any effects of a neutralizing antibody against CD74.

We also explored the effects of DDT on primary epithelial cells by using a model of lung organoids. The first two days of organoid culture are critical for organoid formation and differentiation and after this window organoids will further grow into their chosen differentiation path [34]. For murine organoids, we found DDT to stimulate alveolar differentiation specifically and not airway differentiation. Since ACKR3 appears to be expressed on both airway epithelial cells as well as alveolar epithelial cells in mouse lung tissue (Supplemental data file 1 Fig. 9), this may also point at an unknown interaction with another receptor more specifically expressed on alveolar epithelium. It is of interest to note that a recent publication by Rogers and colleagues showed that in comparison to levels measured in healthy adults, MIF and DDT plasma concentrations were higher in fetuses, increased further at birth, reached strikingly higher levels on postnatal day 4, and decreased to adult levels during the first months of life [46]. This supports an important role for these cytokines during the fetal and neonatal period and suggests that MIF/DDT may indeed be involved in lung development in general and alveolarization in particular as their levels peak in the time window important for alveolarization [47]. Further studies will have to look into the specific roles of MIF and DDT during lung development and whether they are redundant or have separate functions.

We did not find dedicated airway epithelial organoids in cultures from human cells like we did in for mouse cells. The reason for this difference is unclear but may be caused by the underlying disease, i.e. organoids derived from patients with COPD. Importantly, all organoids had ATII cells and we found more and bigger organoids after DDT treatment, indicating that DDT can still induce alveolar growth in organoids from COPD patients. Impaired alveolar repair is a characteristic of emphysematous COPD and a significant clinical problem that cannot be treated pharmacologically at present. Therefore, our finding that DDT can contribute to alveolar growth in organoids derived from COPD patients is interesting and should be further investigated for its relevance to the clinical situation of patients with emphysema. However, careful consideration should be given to the fact that DDT has also been shown to have pro-tumerogenic potential which may complicate its therapeutic use [48], [49], [50].

In conclusion, we found a new function of DDT in lung tissue repair and identified a role for ACKR3 in lung tissue. DDT contributes to alveolar growth, even in organoids derived from lung tissue of COPD patients and should therefore be further investigated as a potential inhibitor of alveolar tissue loss in COPD.

## Author contributions

Shanshan Song: Study design, collection and assembly of data, data analysis and interpretation, manuscript writing, critical reading and revision.

Bin Liu: Literature search, collection and assembly of data, experimental material support, critical reading and revision.

Habibie: Collection and assembly of data, data analysis and interpretation, critical reading and revision.

Jelle Van den Bor: Study design, collection and assembly of data, data analysis and interpretation, critical reading and revision.

Martine J. Smit: Study design, critical reading and revision.

Reinoud Gosens: Experimental material support, critical reading and revision.

Xinhui Wu: Collection and assembly of data, experimental material support, critical reading and revision.

Corry-Anke Brandsma: Collection and assembly of data, critical reading and revision.

Hidde J. Haisma: Experimental material support, critical reading and revision.

Robbert. H.Cool: Collection and assembly of data, critical reading and revision.

Gerrit J. Poelarends: Study design, data analysis and interpretation, critical reading and revision, final approval of manuscript.

Barbro N. Melgert: Study design, data analysis and interpretation, financial support, manuscript writing, critical reading and revision, final approval of manuscript.

## Data sharing statement

Data are available upon reasonable request by sending a message to the corresponding author.

## Declaration of Competing Interest

Dr. Gosens reports grants from Chiesi, grants from Aquilo, grants from Boehringer Ingelheim, grants from Novartis, outside the submitted work. Dr. Melgert reports grants from Boehringer Ingelheim, outside the submitted work. The other authors have no competing interests to declare, financial or otherwise.

## Appendix Supplementary materials

Image, application 1

Image, application 2

Image, application 3

Image, application 4
