# Supplementary material for: D-dopachrome tautomerase contributes to lung epithelial repair via atypical chemokine receptor 3-dependent Akt signaling
Source: eBioMedicine. 2021 Jun 4;68:103412. doi: 10.1016/j.ebiom.2021.103412 (PMC8185224; doi:10.1016/j.ebiom.2021.103412)
Supplement: Supplementary file 1 [file mmc1.docx]

1. Supplemental data file 1
2. Supplemental data file 2
