# Supplementary material for: D-dopachrome tautomerase contributes to lung epithelial repair via atypical chemokine receptor 3-dependent Akt signaling
Source: eBioMedicine. 2021 Jun 4;68:103412. doi: 10.1016/j.ebiom.2021.103412 (PMC8185224; doi:10.1016/j.ebiom.2021.103412)
Supplement: Supplementary file 2 [file mmc2.docx]

**Supplemental data file 1 to:**

**D-dopachrome tautomerase contributes to lung epithelial repair via atypical chemokine receptor 3-dependent Akt signaling**

**Shanshan Song^1,2^, Bin Liu^2^, Habibie Habibie^1,3,4^, Jelle van den Bor^5^, Martine J. Smit^5^, Reinoud Gosens^1,3^, Xinhui Wu^1,3^, Corry-Anke Brandsma^3,6^, Robbert. H. Cool^2^, Hidde J. Haisma^2^, Gerrit J. Poelarends^2^, Barbro N. Melgert^1,3^**

*1. Groningen Research Institute of Pharmacy, Department of Molecular Pharmacology, University of Groningen, Antonius Deusinglaan 1, 9713 AV, Groningen, The Netherlands*

*2. Groningen Research Institute of Pharmacy, Department of Chemical and Pharmaceutical Biology, University of Groningen, Antonius Deusinglaan 1, 9713 AV, Groningen, The Netherlands*

*3. University Medical Center Groningen, Groningen Research Institute of Asthma and COPD, University of Groningen, Hanzeplein 1, 9713 GZ, Groningen, The Netherlands*

*4. Faculty of Pharmacy, Hasanuddin University,* *Makassar 90245, Indonesia*

*5. Division of Medicinal Chemistry, Amsterdam Institute of Molecular and Life Sciences, Vrije Universiteit Amsterdam, De Boelelaan 1108, 1081 HZ, Amsterdam, The Netherlands*

*6. University Medical Center Groningen, Department of Pathology and Medical Biology, University of Groningen, Hanzeplein 1, 9713 GZ Groningen*


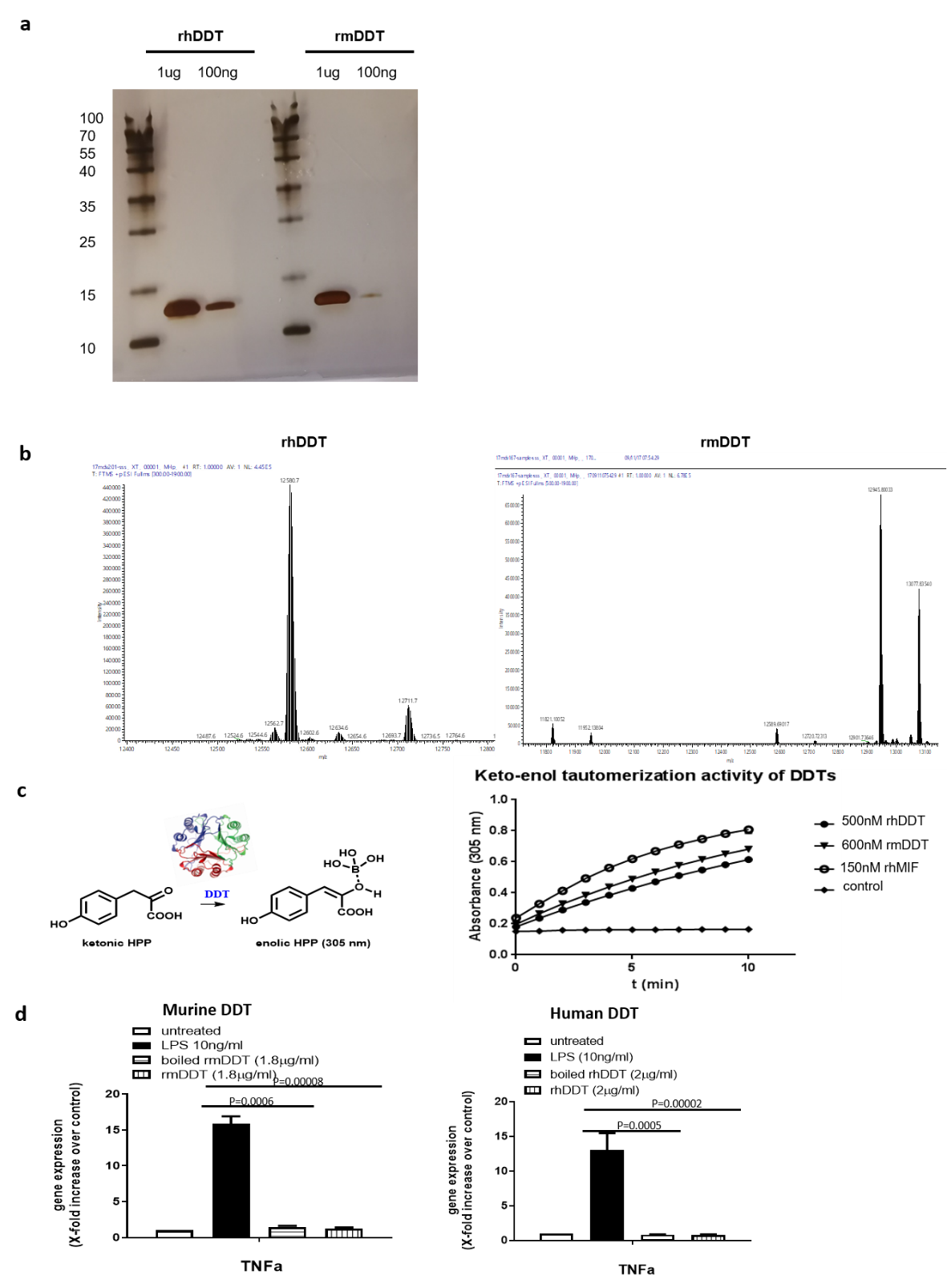


**Figure 1. Recombinant murine and human DDT have tautomerase activity and do not induce TNFα expression in murine RAW264.7 macrophages.**

(a) Silver staining of purified human (left part of the blot) and murine DDT (right part of the blot). The first lane shows the protein molecular weight markers. 1µg and 100ng of purified human or murine DDT were loaded on the gel respectively. (b) Mass spectroscopy of rhDDT and rmDDT.(c) The mechanism of 4-HPP tautomerization assay and results. The enzymes rhDDT and rmDDT were diluted in boric acid buffer (435 mM, pH 6∙2) to a concentration of 1110 nM for rhDDT and 1330 nM for rmDDT, respectively. Next, 180 µl diluted DDT was mixed with 10 µl EDTA/Tween20 buffer and 10 µl DMSO to make a final solution including 1∙0 mM EDTA, 0∙02% (v/v) Tween20, 5%(v/v) DMSO and corresponding enzyme. The reaction was started by adding 50 µl of the mixture to 50 µl 4-HPP solution (1 mM in pH 6∙0 50 mM ammonium acetate buffer). Product was detected by monitor the UV absorbance at 305 nm. MIF was applied as reference. A negative control was included in which no enzyme was added. (d) RAW264.7 macrophages were stimulated with LPS (10 ng/ml), boiled rmDDT(1.8 µg/ml), rmDDT (1.8 µg/ml), boiled rhDDT (2 µg/ml), or rhDDT (2 µg/ml). Relative TNFα mRNA expression was measured by qPCR.


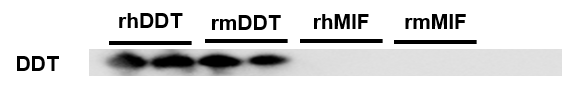


**Figure 2**. **The antibody against DDT does not cross react with MIF.**

2ug of rhDDT, rmDDT, rhMIF or rmMIF was loaded onto a gel and transferred to a PVDF membrane. The membranes were incubated overnight at 4 °C with an antibody against DDT. Blotted proteins were visualized with an ECL^TM^ Prime Western Blotting System. Original images can be found in supplemental data file 2.

**
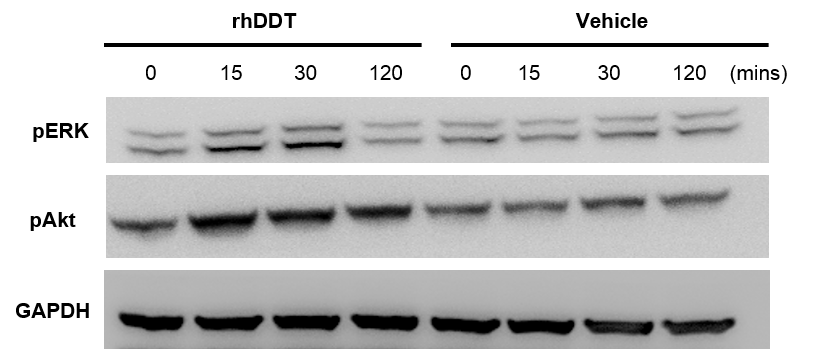
**

**Figure 3**. A v**ehicle control has no effect on phosphorylation of ERK and Akt, whereas DDT induces phosphorylation of both proteins.**

A549 epithelial cells were treated with 100 ng/ml rhDDT for different time periods. Cell lysates were analyzed for phosphorylation of ERK (pERK) and Akt (pAkt). GAPDH was used as a loading control. Original images can be found in supplemental data file 2.


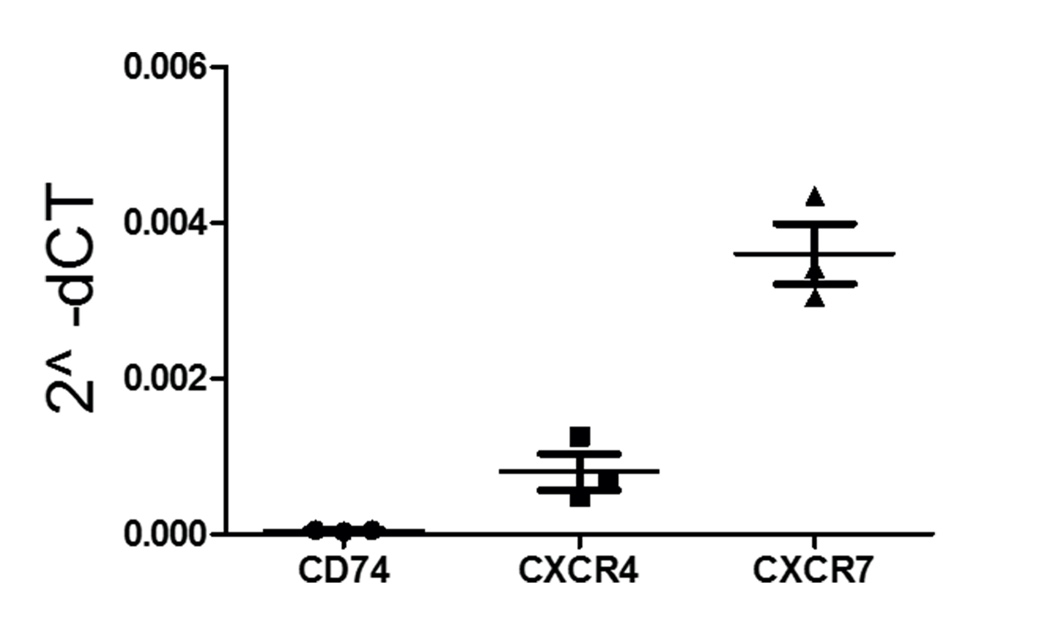


**Figure 4:** **CD74, CXCR4, ACKR3 mRNA expression in A549 epithelial cells.**

Relative mRNA expression of CD74, CXCR4 and ACKR3 In A549 epithelial cells as measured by qPCR.

**
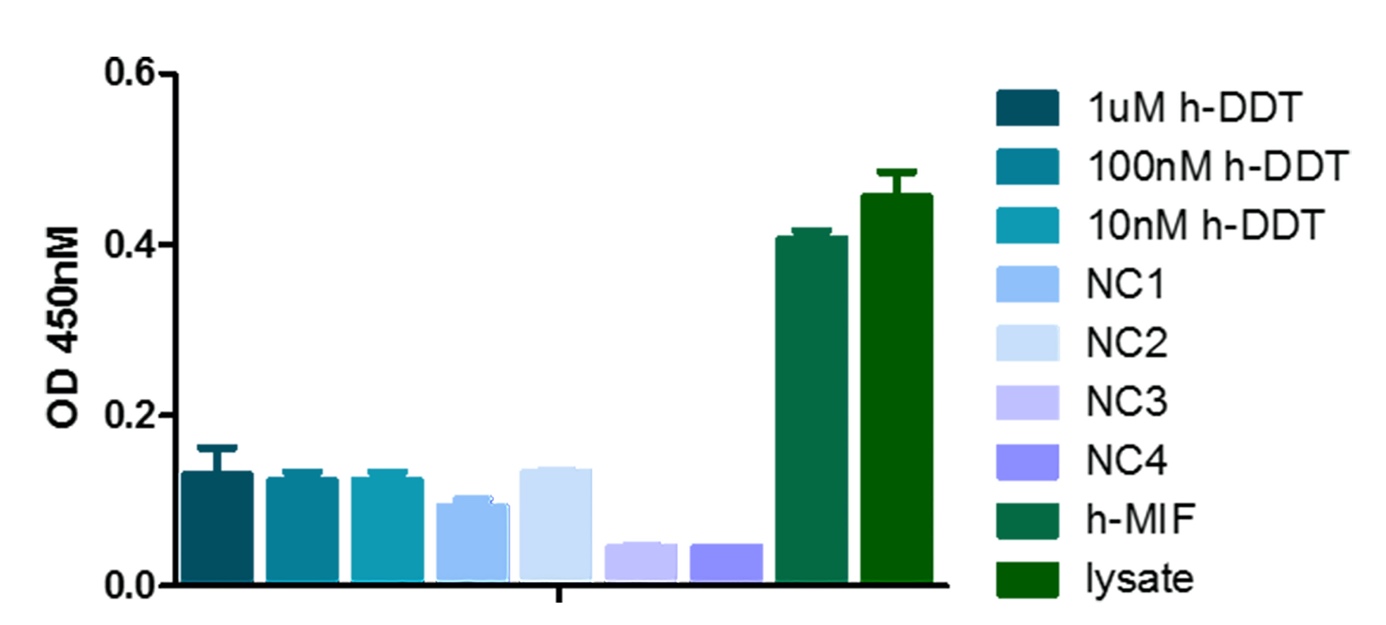
**

**Figure 5. No evidence of binding between DDT and CXCR4 using ELISA.**

Binding of DDT to CXCR4 was measured by ELISA. Wells were coated with DDT and binding of CXCR4-containing HeLa cell lysate was lower than in wells coated with MIF or with CXCR4-containing HeLa cell lysate directly. In fact, binding was comparable to the negative controls. Negative controls (NC) assessed aspecific binding between rhDDT and the primary antibody (no CXCR4-containing HeLa cell lysate added, NC1), aspecific binding between CXCR4 and the secondary antibody (no primary antibody added, NC2), BSA coating added instead of rhDDT (NC3), and lysis buffer added instead of HeLa cell lysate (NC4). Each experiment was performed in triplicate and three independent experiments were done.


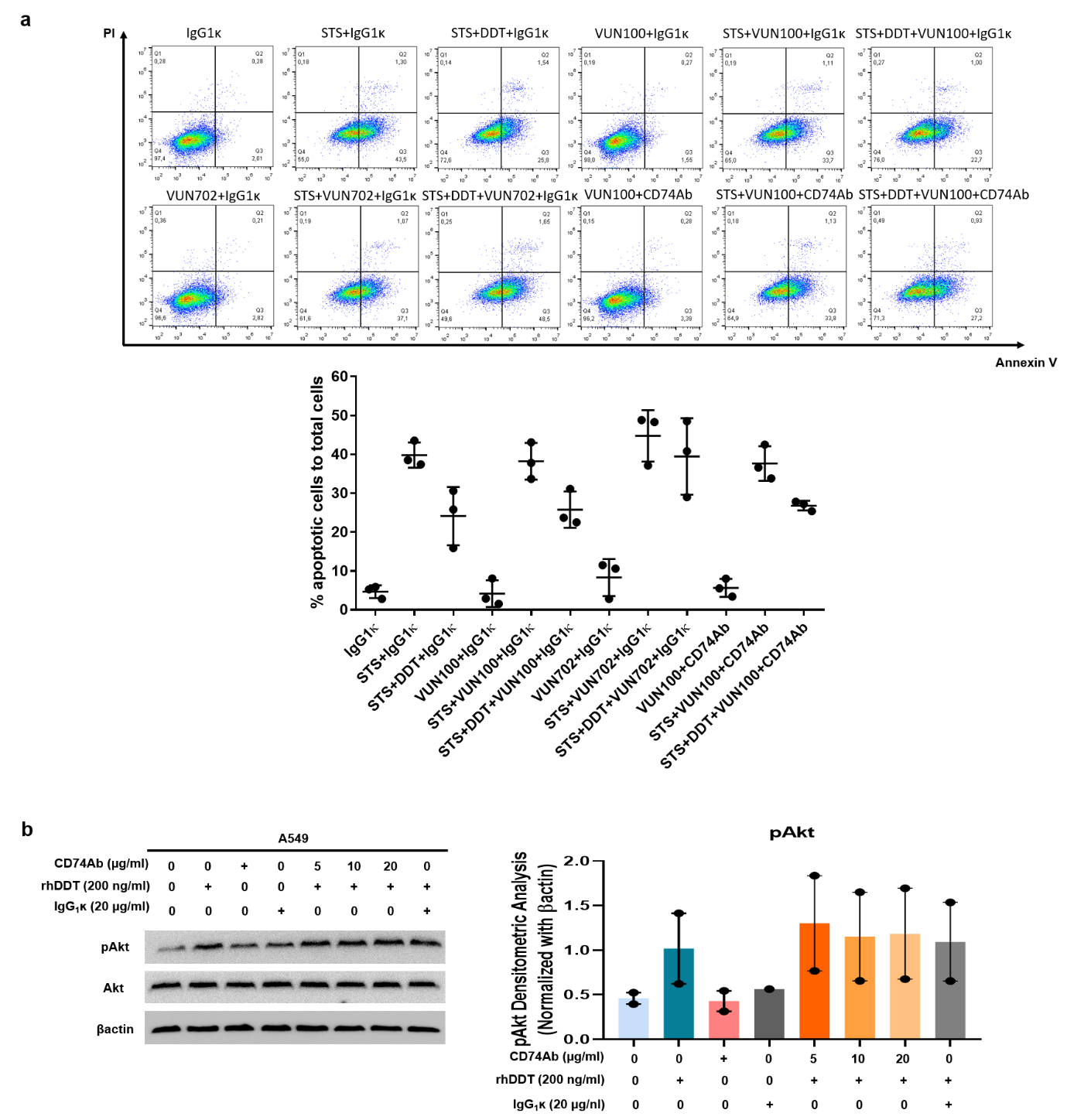


**Figure 6. A neutralizing antibody against CD74 did not inhibit DDT-induced effects.**

(a) A549 epithelial cells were pretreated with 20 µg/ml isotype control IgG_1Ƙ_ or 1 μM of control nanobody VUN100 prior to treatment with 100 ng/ml DDT. Then cells were treated with staurosporine for 24 h. Three independent experiments were done. (b) A549 epithelial cells were pretreated with different doses of CD74 antibody prior to treatment with 200 ng/ml DDT for 15mins. Cell lysates were analyzed for phosphorylation of Akt (pAkt) by western blot (n=2, original images can be found in supplemental data file 2).


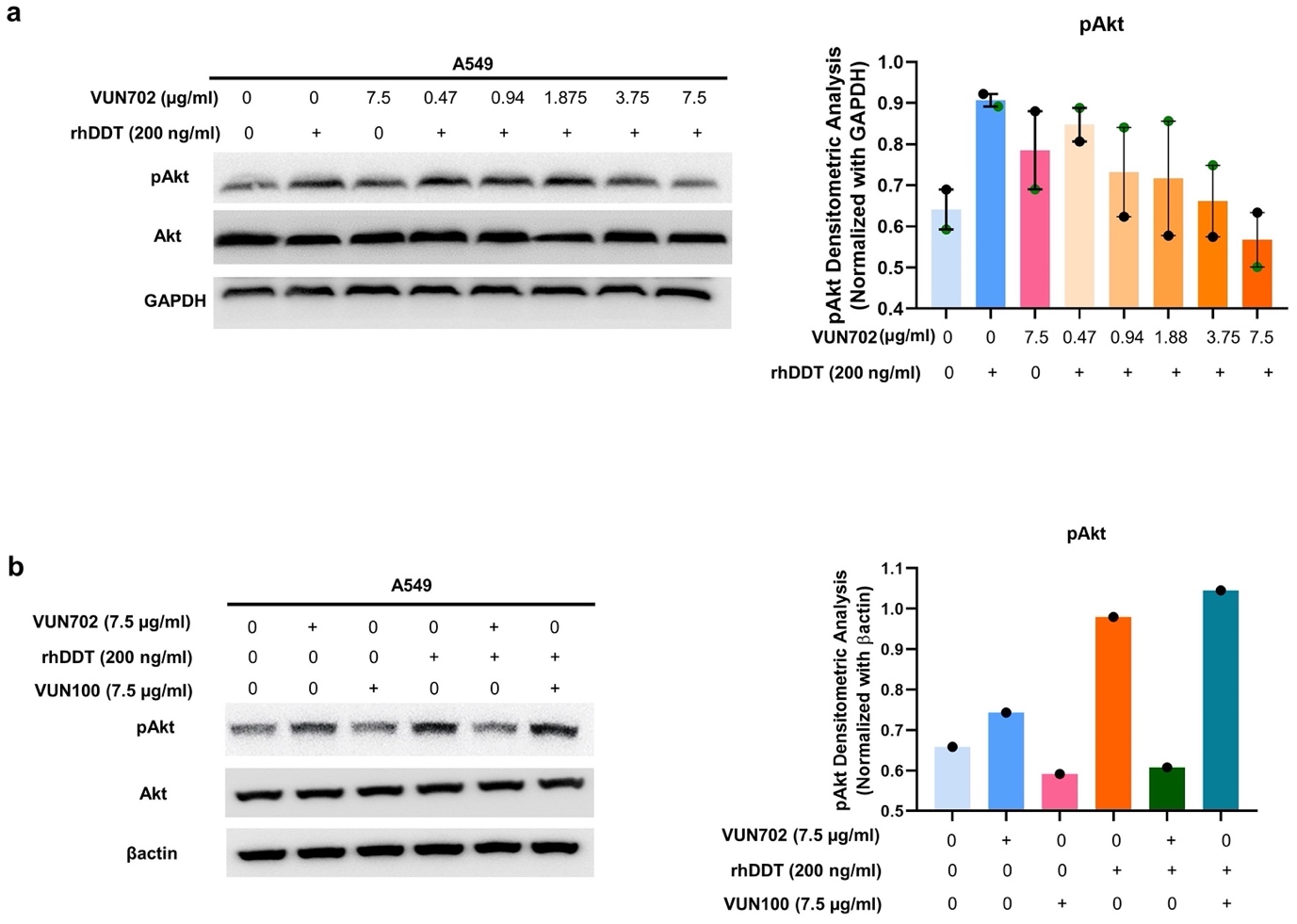


**Figure 7. The ACKR3 nanobody VUN702 inhibits DDT-induced phosphylation of Akt.**

(a) A549 epithelial cells were pretreated with different doses of VUN702 prior to treatment with 200 ng/ml DDT for 15mins. Cell lysates were analyzed for -pAkt by western blot (n=2, original images can be found in supplemental data file 2). (b) A549 epithelial cells were pretreated with VUN702 (7.5 µg/ml) and control nanobody VUN100 (7.5 µg/ml) prior to treatment with 200 ng/ml DDT for 15mins. Cell lysates were analyzed for pAkt by western blot (n=1, original image can be found in supplemental data file 2).


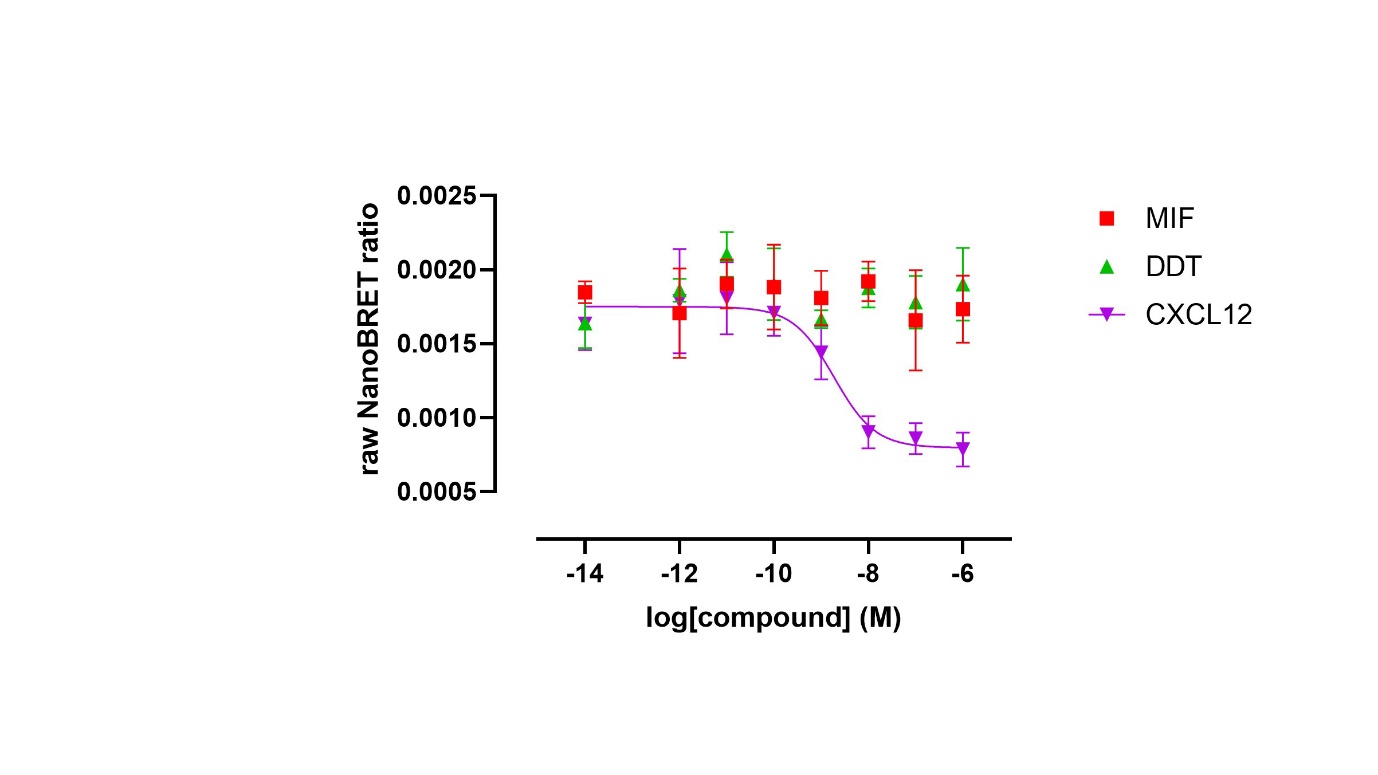


**Figure 8. DDT and MIF do not displace CXCL12-AF647 from ACKR3, but unlabeled CXCL12 does.**

Binding of DDT, MIF or CXCL12 to Nluc-ACKR3 was measured using NanoBRET. A549 epithelial cells were transfected with Nluc-ACKR3 using lipofectamine 3000. Two days after transfection, 3 nM of CXCL12-AF647 was coincubated with unlabeled MIF, DDT or CXCL12 for 2 h at 4°C to prevent internalization. After 2 h, NanoGlo® was added after which the excitation of Nluc (460-80 nm) and CXCL12-AF647 (610-LP nm) was measured.

**
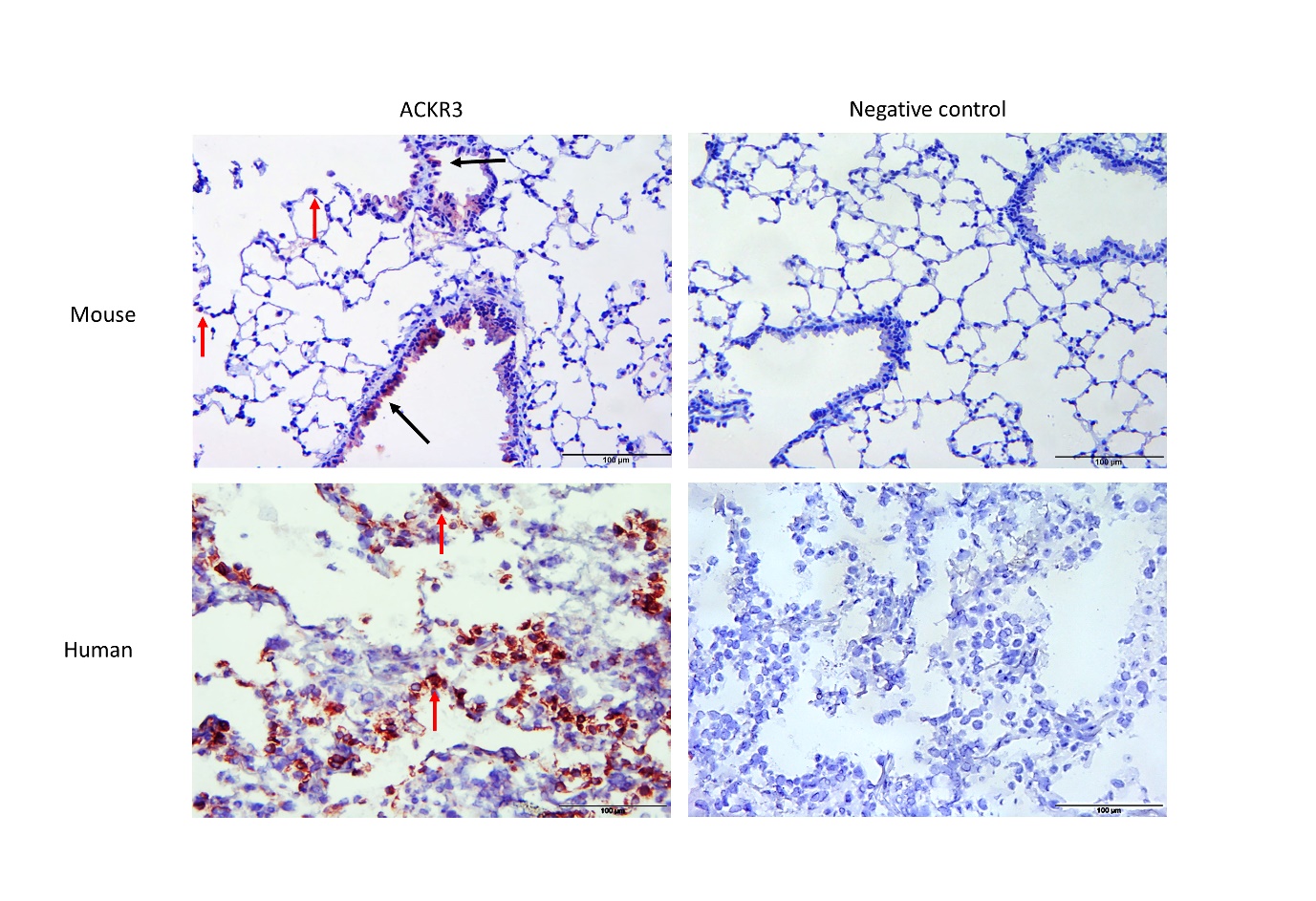
**

**Figure 9. ACKR3 protein is expressed in control human and mouse lung tissue.**

Representative pictures of an ACKR3 staining in normal lung tissue from a patient undergoing surgical resection for lung cancer (upper left panel) and lung tissue from a healthy mouse (lower left panel). AKCR3-specific staining was observed in alveolar cells (red arrows) and ciliated epithelial cells (black arrows) as indicated by the arrows. Right panels display negative control stainings.
