# Supplementary material for: D-dopachrome tautomerase contributes to lung epithelial repair via atypical chemokine receptor 3-dependent Akt signaling
Source: eBioMedicine. 2021 Jun 4;68:103412. doi: 10.1016/j.ebiom.2021.103412 (PMC8185224; doi:10.1016/j.ebiom.2021.103412)

Supplemental data file 2 to:

D-dopachrome tautomerase contributes to lung epithelial repair via atypical chemokine receptor 3-dependent Akt signaling

Shanshan Song[1,2], Bin Liu[2], Habibie Habibie[1,3,4], Jelle van den Bor[5], Martine J. Smit[5], Reinoud Gosens[1,3], Xinhui Wu[1,3], Corry-Anke Brandsma[3,6], Robbert. H. Cool[2], Hidde J. Haisma[2], Gerrit J. Poelarends[2], Barbro N. Melgert[1,3]

1. Groningen Research Institute of Pharmacy, Department of Molecular Pharmacology, University of Groningen, Antonius Deusinglaan 1, 9713 AV, Groningen, The Netherlands

2. Groningen Research Institute of Pharmacy, Department of Chemical and Pharmaceutical Biology, University of Groningen, Antonius Deusinglaan 1, 9713 AV, Groningen, The Netherlands

3. University Medical Center Groningen, Groningen Research Institute of Asthma and COPD, University of Groningen, Hanzeplein 1, 9713 GZ, Groningen, The Netherlands

4. Faculty of Pharmacy, Hasanuddin University, Makassar 90245, Indonesia

5. Division of Medicinal Chemistry, Amsterdam Institute of Molecular and Life Sciences, Vrije Universiteit Amsterdam, De Boelelaan 1108, 1081 HZ, Amsterdam, The Netherlands

6. University Medical Center Groningen, Department of Pathology and Medical Biology, University of Groningen, Hanzeplein 1, 9713 GZ Groningen

Figure 3a

Replicate 1

Replicate 2

Replicate 3

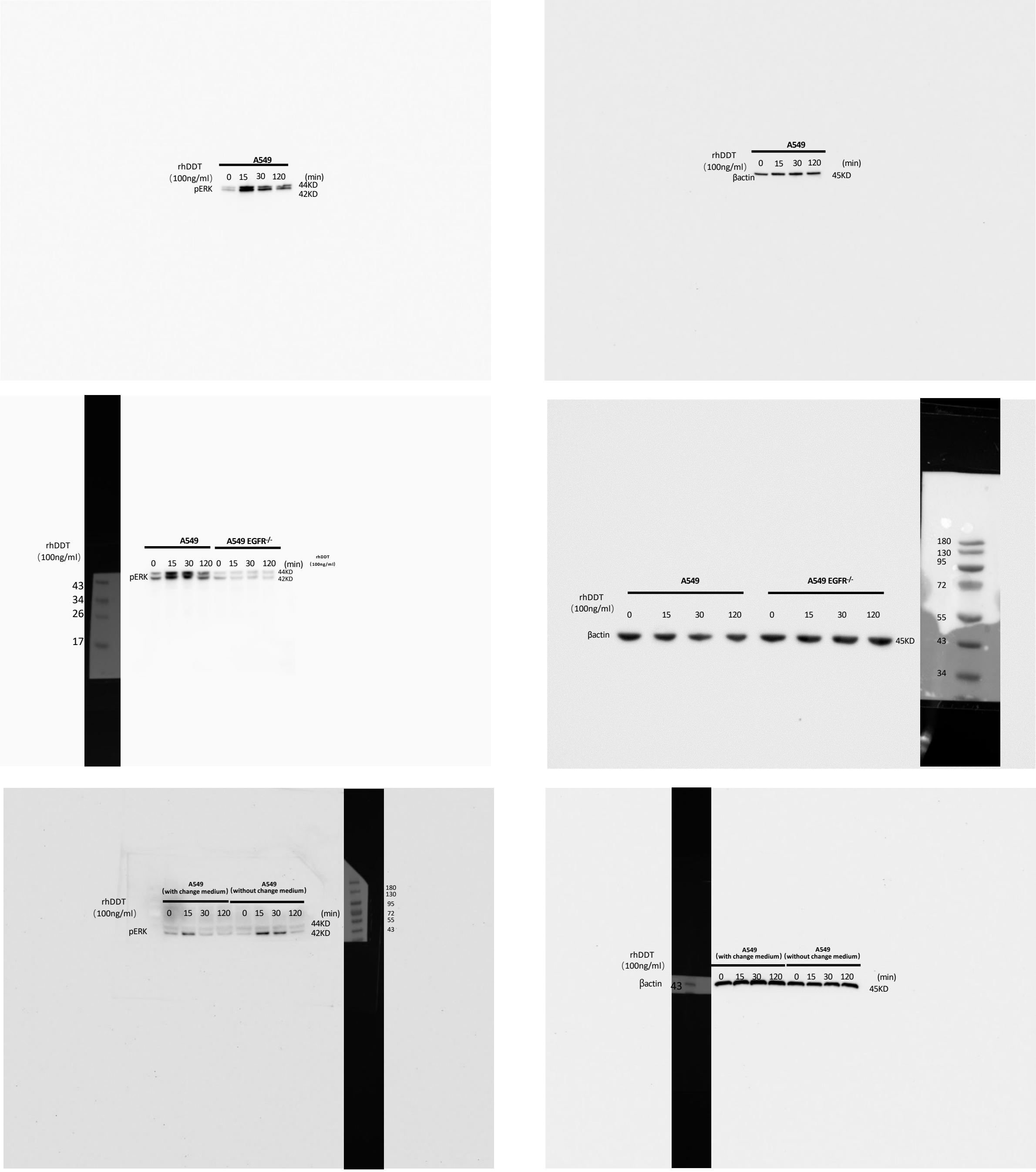

We used the data of "without change medium" for the manuscript.

Figure 3b

Replicate 1

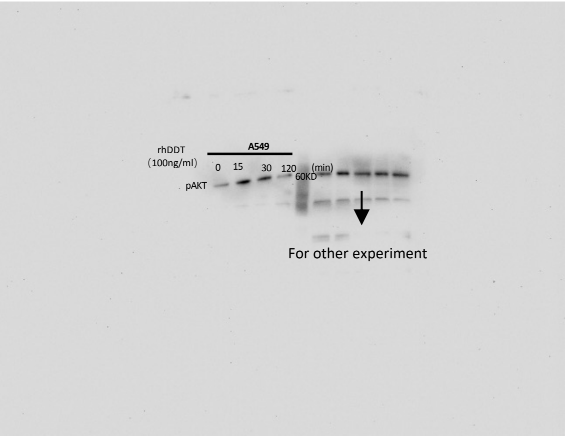

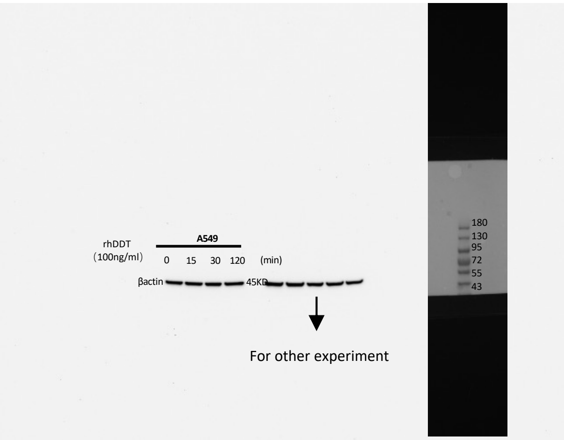

Replicate 2

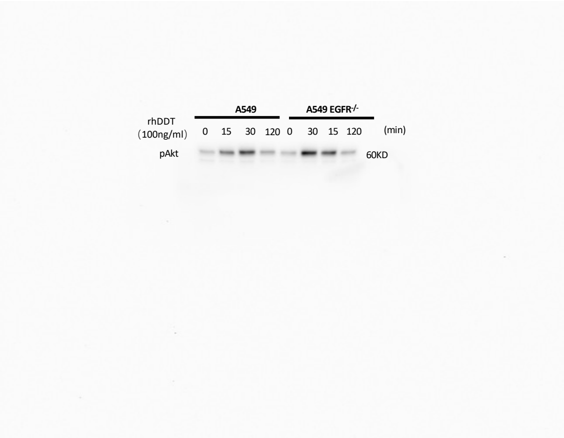

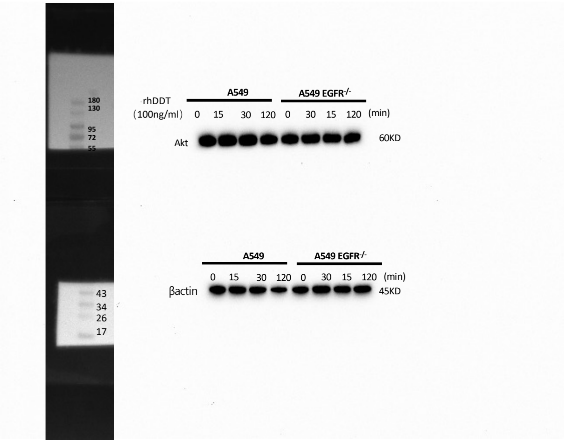

We used parts of the data from this experiment for figure 3b/d and figure 4g. In figure 3 we used results from the wildtype A549 cells and in figure 4g we compared wildtype A549 cells to EGFR KO A549 cells.

Replicate 3

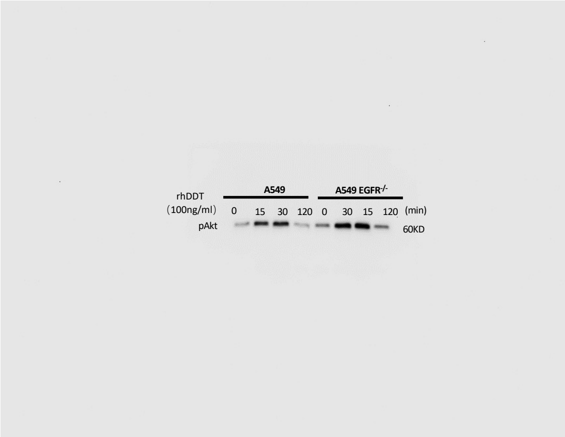

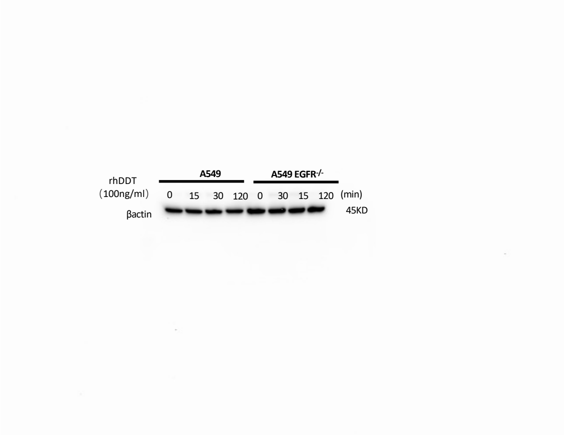

Figure 3d

Replicate 1

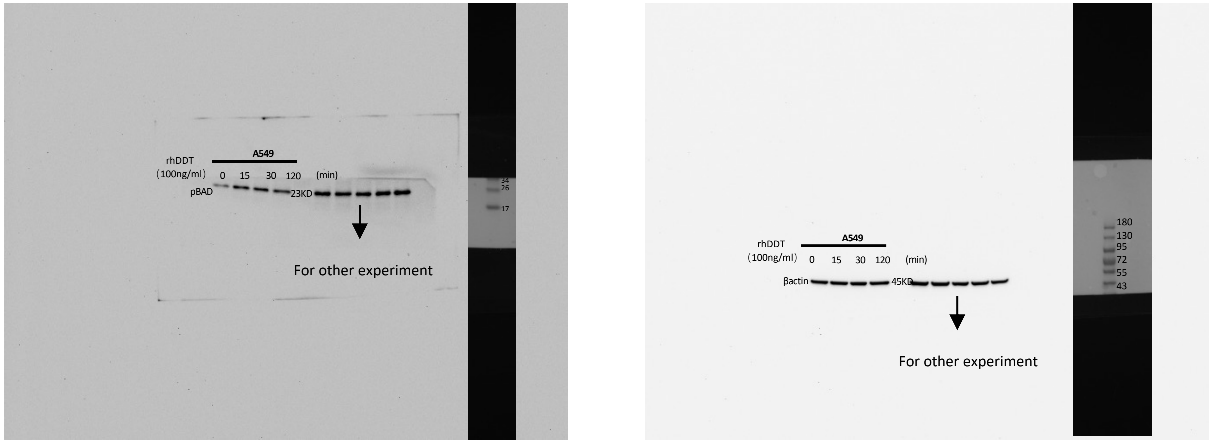

Replicate 2

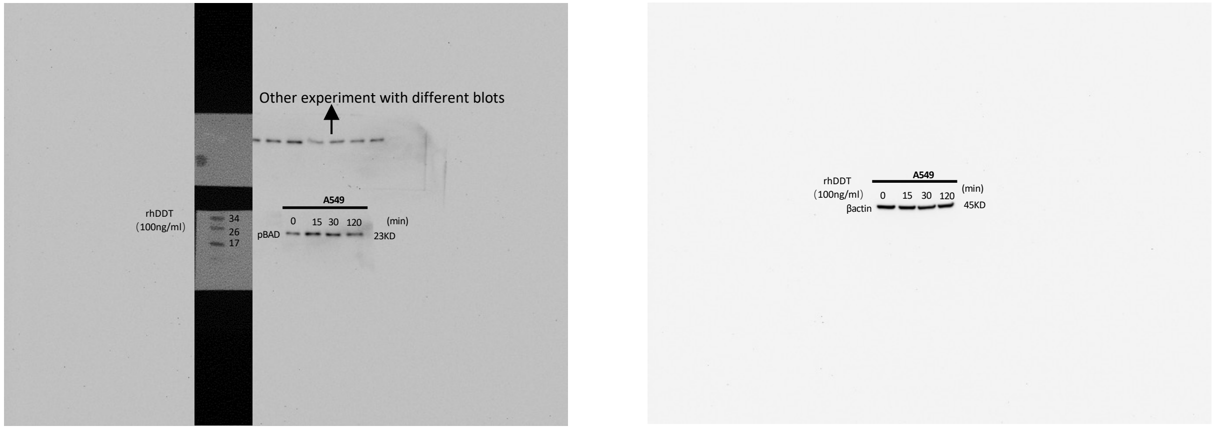

Replicate 3

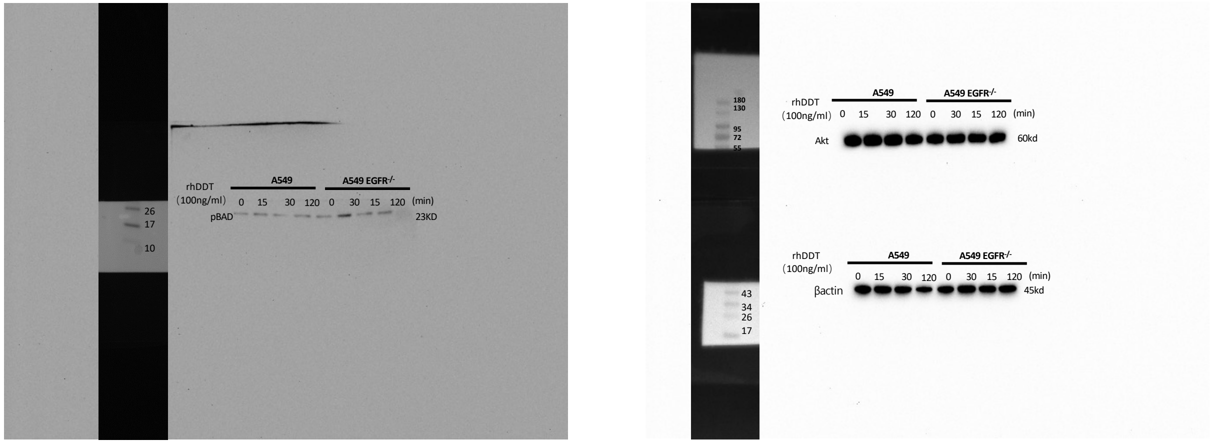

Figure 4b

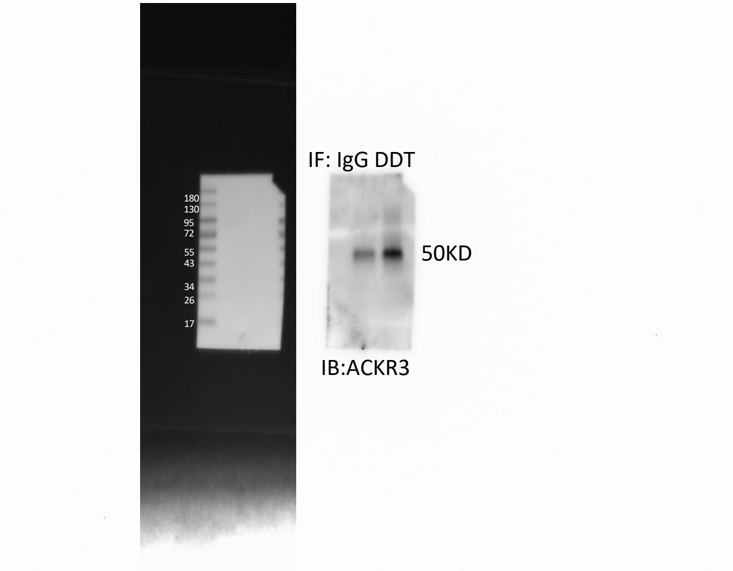

180
130
95
72
55
43
34
26
17
IF: IgG DDT
50KD
IB:ACKR3

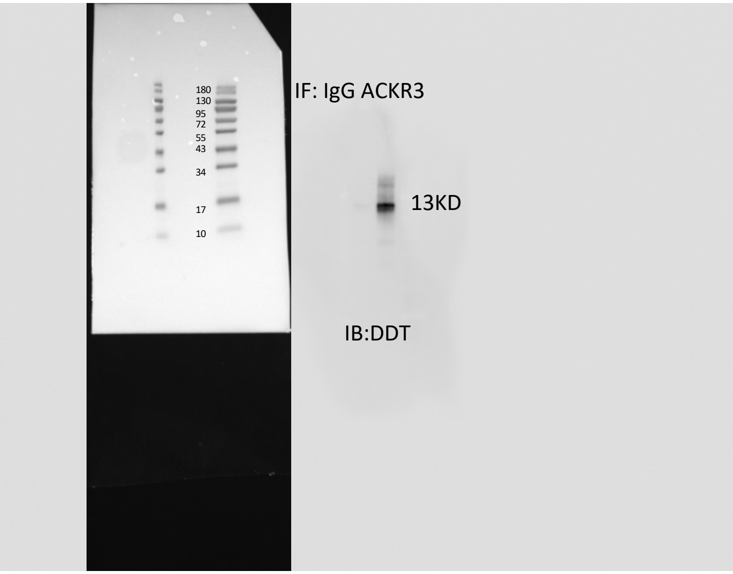

180
130
95
72
55
43
34
17
10
IF: IgG ACKR3
13KD
IB:DDT

Figure 4g
Replicate 1

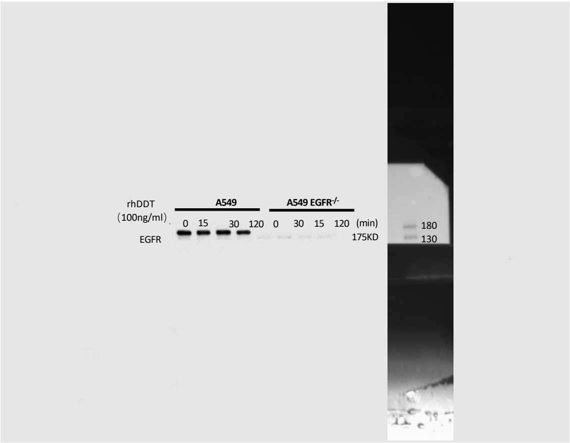

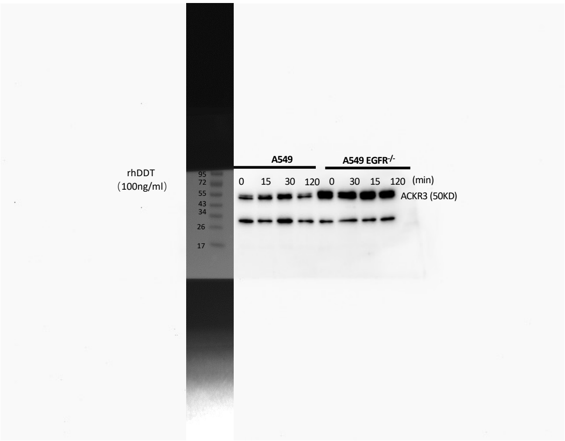

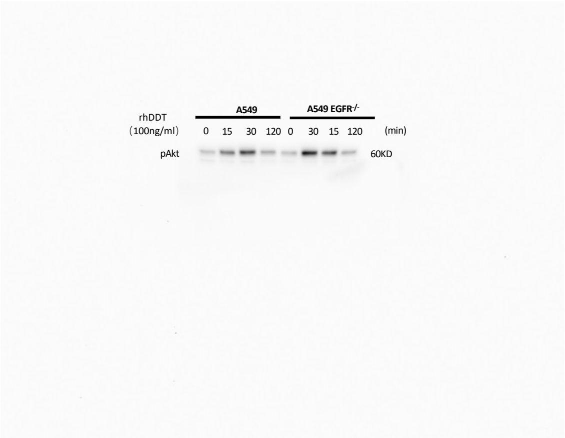

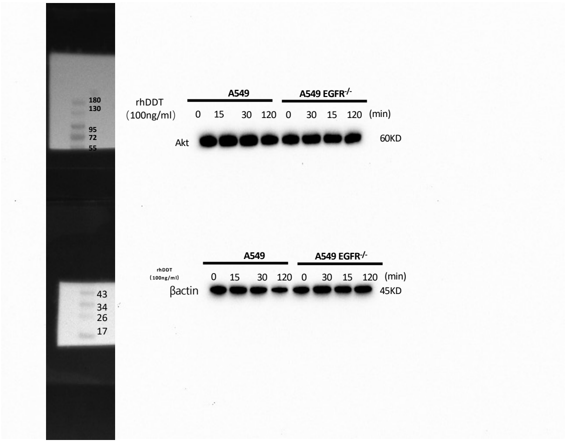

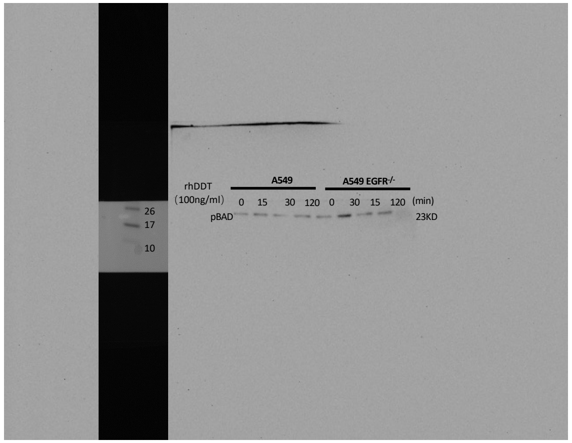

Figure 4g
Replicate 2

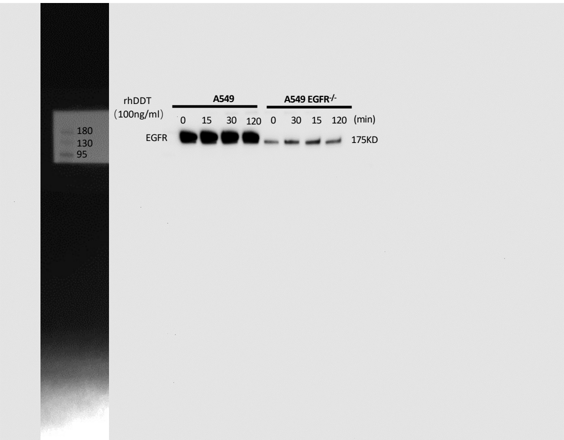

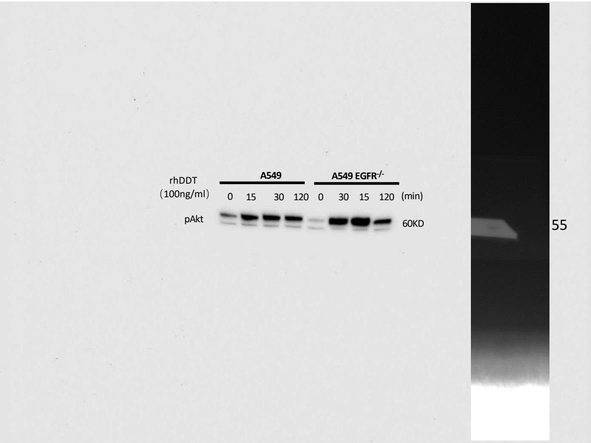

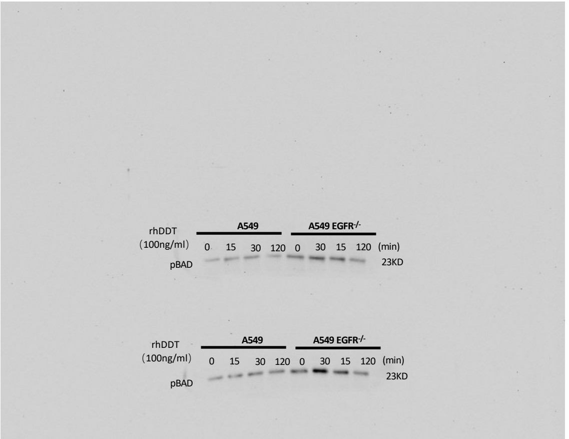

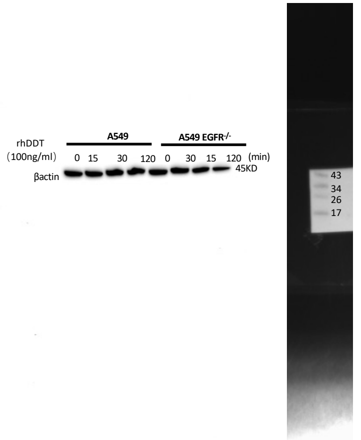

Figure 4g
Replicate 3

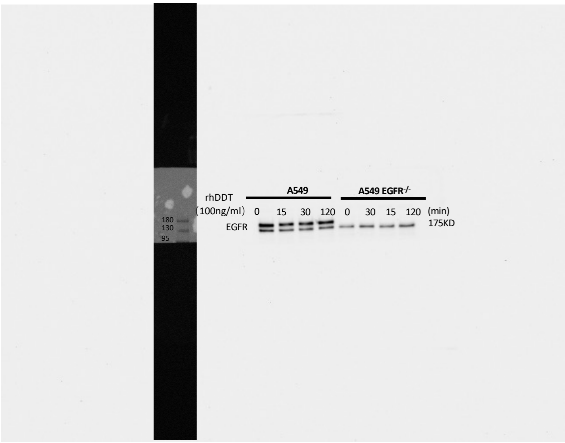

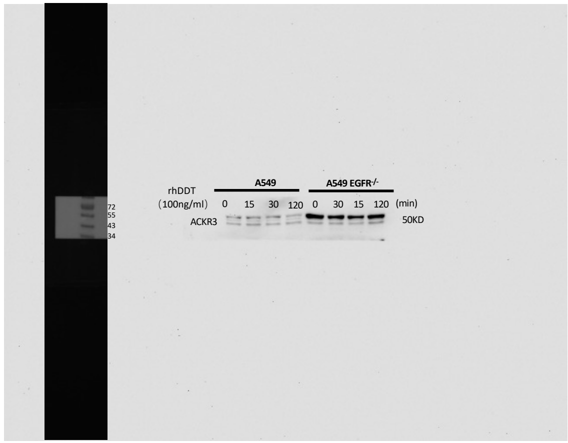

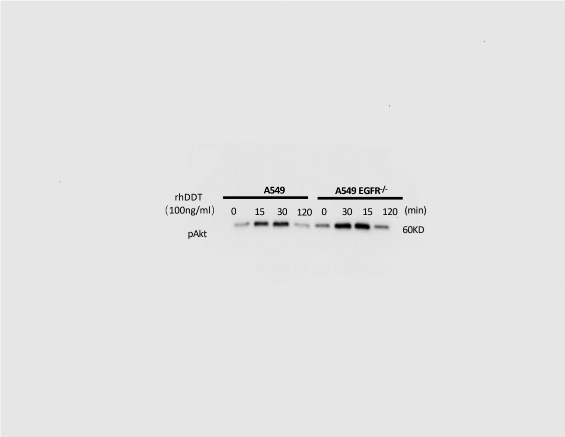

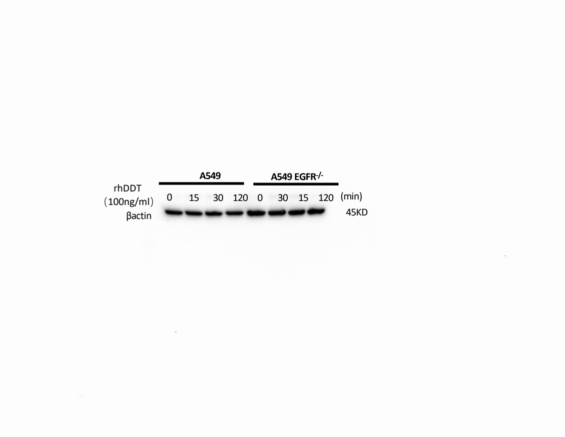

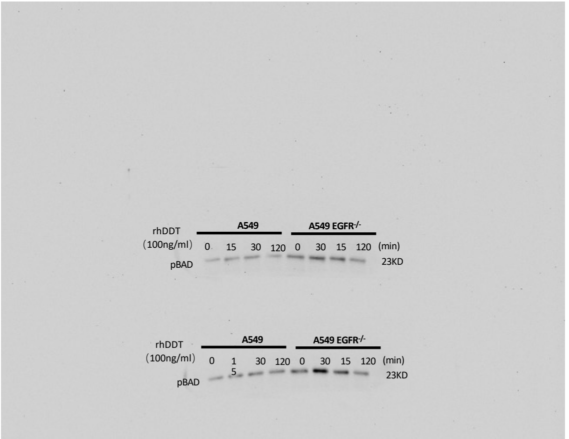

Supplementary figure 2

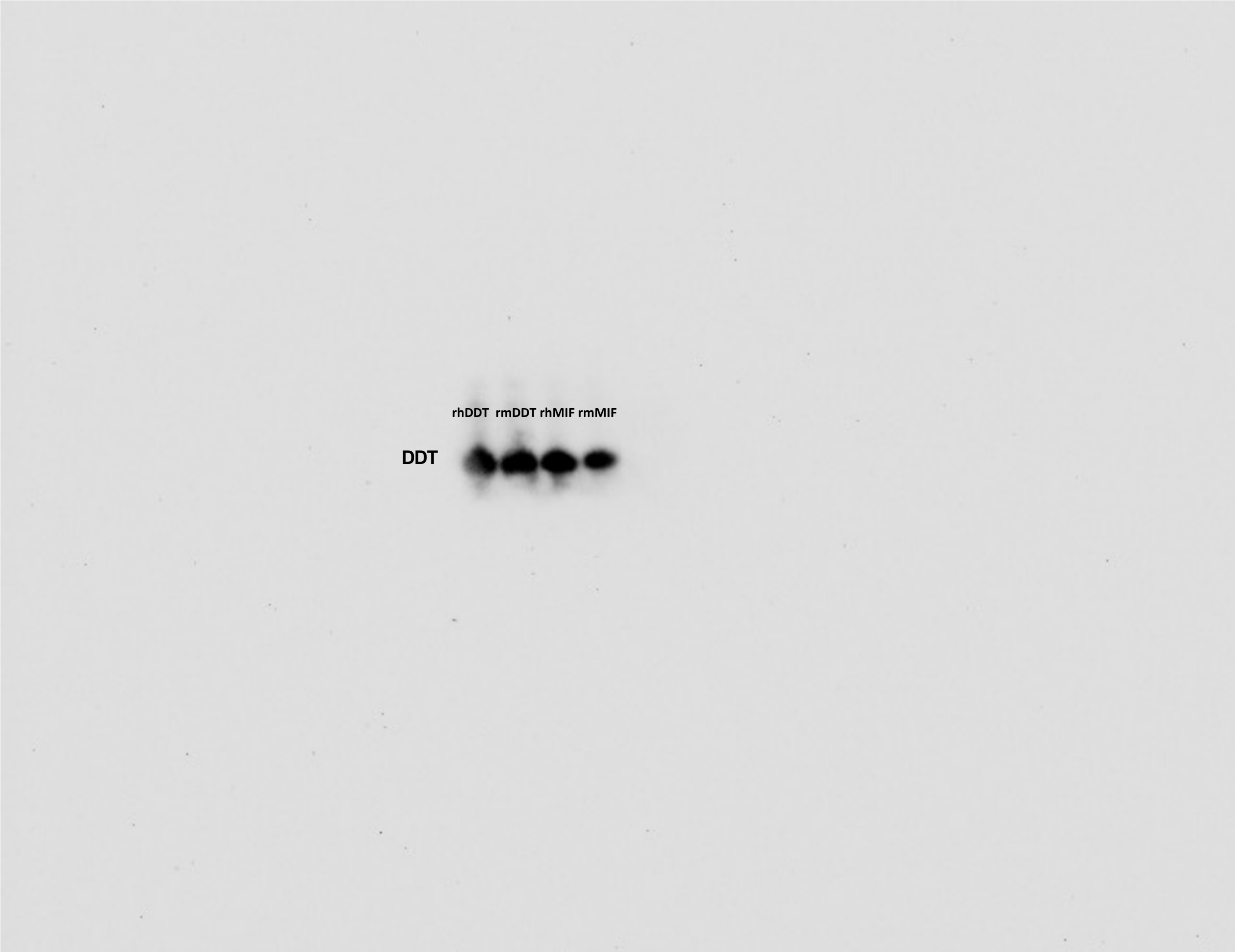

Supplementary figure 3

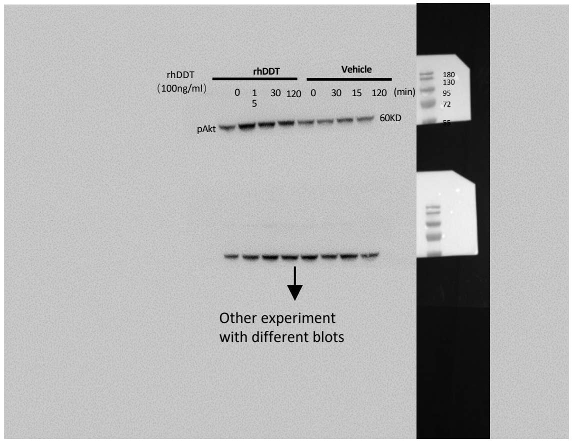

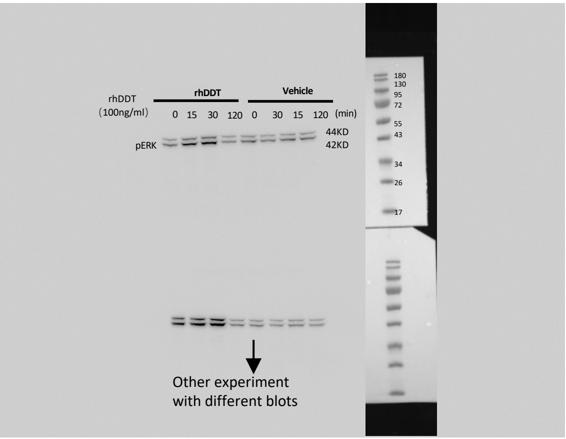

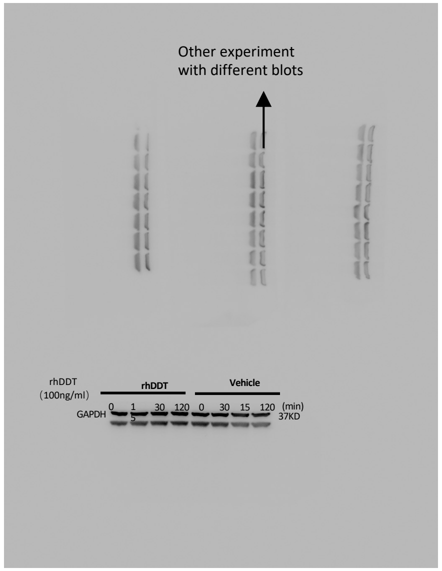

Supplementary figure 6b

Replicate 1

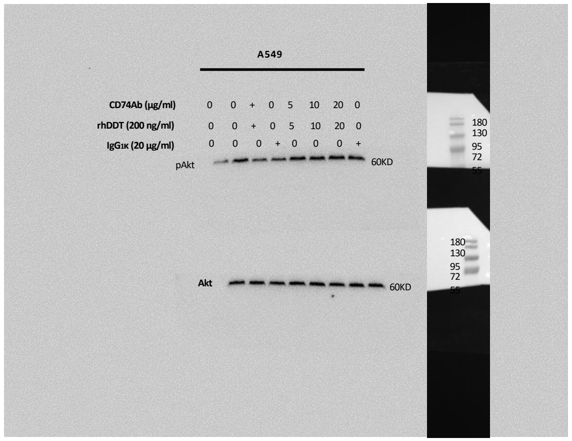

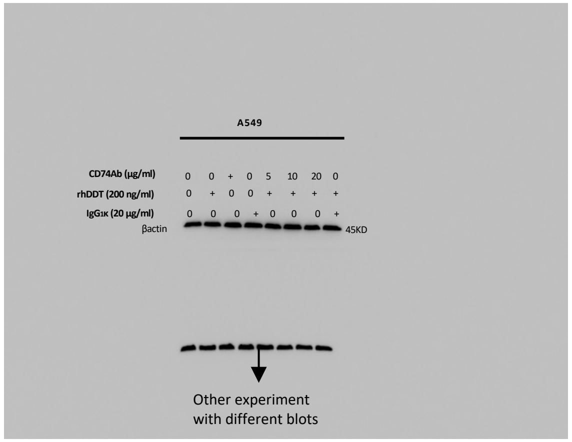

Replicate 2

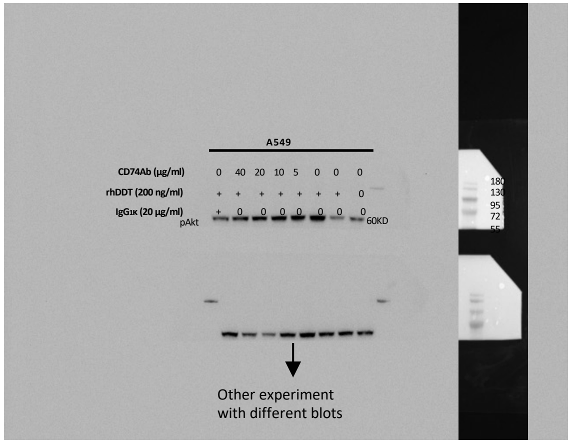

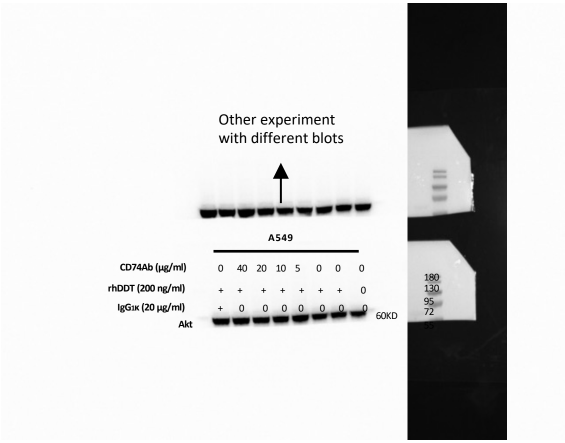

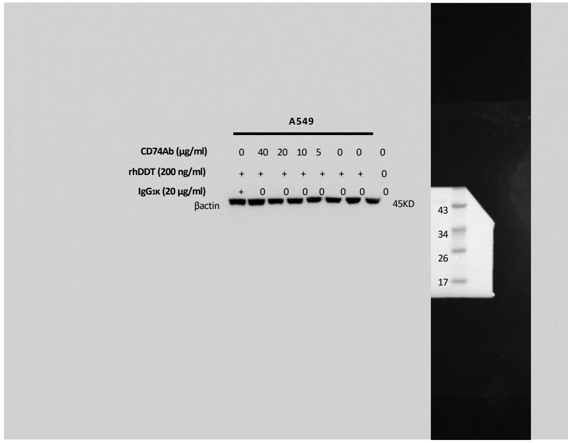

Supplementary figure 7a
Replicate 1

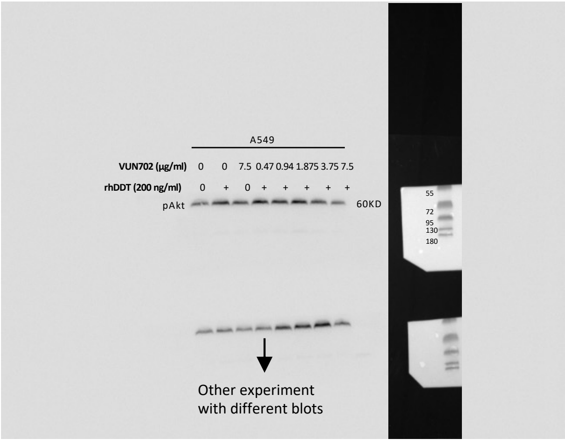

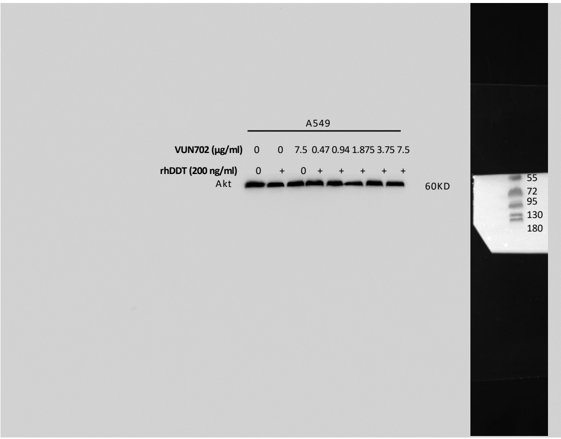

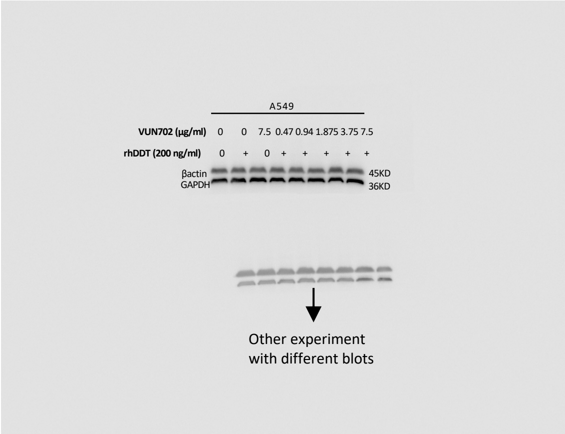

Supplementary figure 7a
Replicate 2

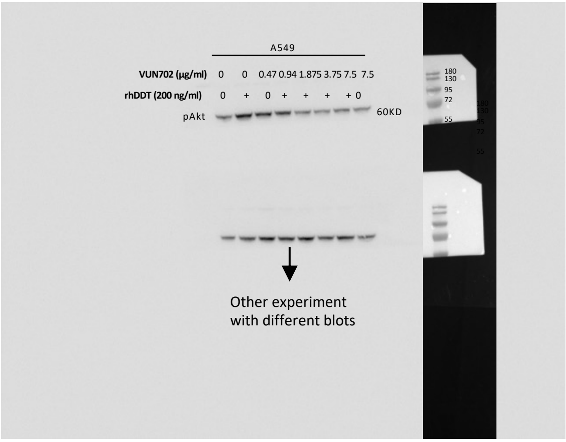

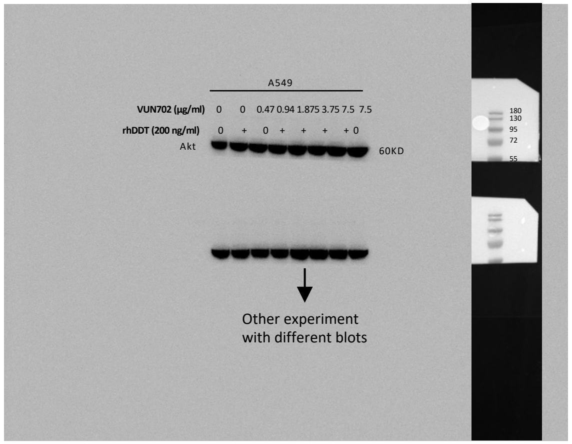

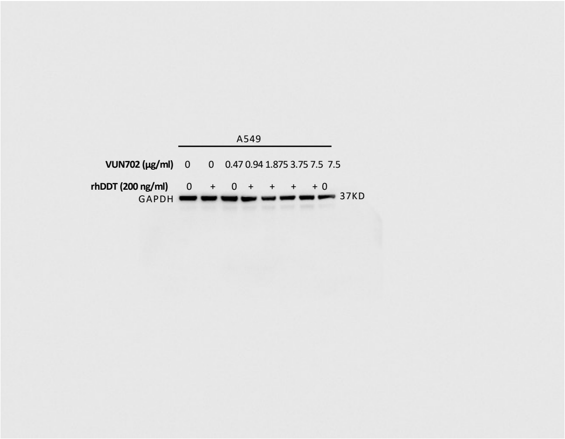

Supplementary figure 7b

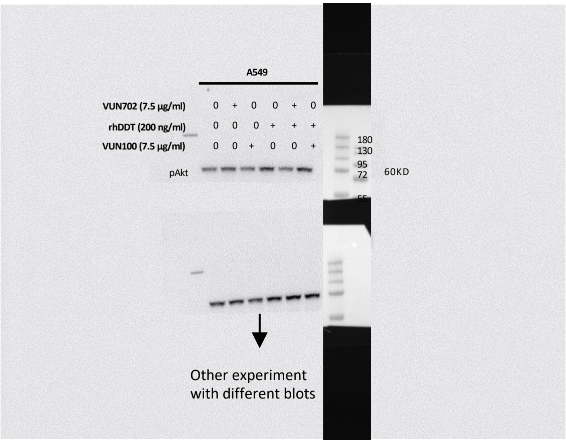

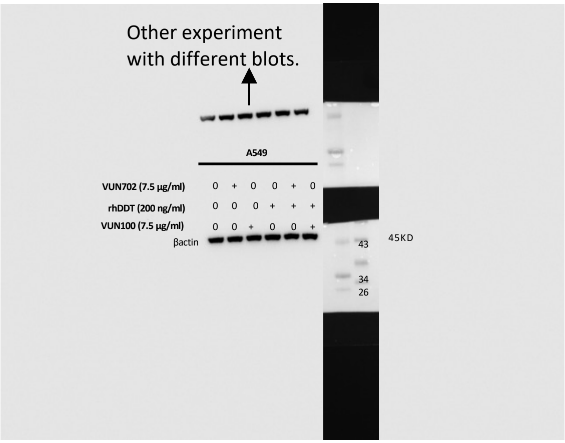

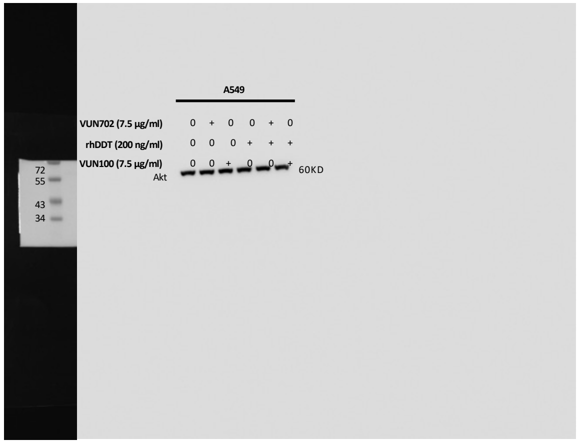

Supplement: Supplementary file 3 [file mmc3.pdf]
