# Supplementary material for: D-dopachrome tautomerase contributes to lung epithelial repair via atypical chemokine receptor 3-dependent Akt signaling
Source: eBioMedicine. 2021 Jun 4;68:103412. doi: 10.1016/j.ebiom.2021.103412 (PMC8185224; doi:10.1016/j.ebiom.2021.103412)
Supplement: Supplementary file 4 [file mmc4.pdf]

Consent for Acknowledgments

Manuscript Title: D-dopachrome tautomerase contributes to lung epithelial repair via atypical chemokine receptor 3-dependent Akt signalling

Corresponding author: Barbro N. Melgert

Article type: Research Article

<u>Person or entity being acknowledged to fill in items below:</u>

I have seen a version of the manuscript to be submitted/published and I hereby give my consent for my name (or name of entity) to be included in the acknowledgement section in the above-named manuscript for consideration of publication in the *EBioMedicine.* I understand that this signed form will be submitted to the journal with the manuscript as evidence of my consent.

I understand that this manuscript may be published in the *EBioMedicine* and in products derived from the journals. As a result, I understand that the material may be seen by the general public. I understand that I may revoke consent at any time before publication, but once the information has been published revocation of the consent is no longer possible. I understand that I will derive no financial benefit from publication of this paper.

Prof Dr H.W. Frijlink

Name of Person (print name) and/or Entity (print entity name and name of person signing)

11-05-2021

Signature (or signature of the person giving consent on behalf of the person)

Date
